# The nature of interspecific interactions and co‐diversification patterns, as illustrated by the fig microcosm

**DOI:** 10.1111/nph.16176

**Published:** 2019-10-04

**Authors:** Ai‐Ying Wang, Yan‐Qiong Peng, Lawrence D. Harder, Jian‐Feng Huang, Da‐Rong Yang, Da‐Yong Zhang, Wan‐Jin Liao

**Affiliations:** ^1^ State Key Laboratory of Earth Surface Processes and Resource Ecology Ministry of Education Key Laboratory for Biodiversity Science and Ecological Engineering Beijing Normal University Beijing China; ^2^ CAS Key Laboratory of Tropical Forest Ecology Xishuangbanna Tropical Botanical Garden Chinese Academy of Sciences Kunming China; ^3^ Department of Biological Sciences University of Calgary 2500 University Drive NW Calgary AB Canada

**Keywords:** co‐diversification, competition, co‐speciation, fig, fig wasp, host switching, mutualism, parasitism

## Abstract

- Interactions between mutualists, competitors, and antagonists have contrasting ecological effects that, sustained over generations, can influence micro‐ and macroevolution. Dissimilar benefits and costs for these interactions should cause contrasting co‐diversification patterns between interacting clades, with prevalent co‐speciation by mutualists, association loss by competitors, and host switching by antagonists.
- We assessed these expectations for a local assemblage of 26 fig species (Moraceae: *Ficus*), 26 species of mutualistic (pollinating), and 33 species of parasitic (galling) wasps (Chalcidoidea). Using newly acquired gene sequences, we inferred the phylogenies for all three clades. We then compared the three possible pairs of phylogenies to assess phylogenetic congruence and the relative frequencies of co‐speciation, association duplication, switching, and loss.
- The paired phylogenies of pollinators with their mutualists and competitors were significantly congruent, unlike that of figs and their parasites. The distributions of macroevolutionary events largely agreed with expectations for mutualists and antagonists. By contrast, that for competitors involved relatively frequent association switching, as expected, but also unexpectedly frequent co‐speciation. The latter result likely reflects the heterogeneous nature of competition among fig wasps.
- These results illustrate the influence of different interspecific interactions on co‐diversification, while also revealing its dependence on specific characteristics of those interactions.

## Introduction

Pairs of species can interact in manners that are mutually beneficial (mutualism), mutually detrimental (competition), or beneficial to one partner but detrimental to the other (antagonism: predation, herbivory, parasitism). If these interactions occur frequently and significantly influence individual performance, then mutualism, competition and antagonism should promote contrasting evolutionary responses (Yoder & Nuismer, 2010; Hembry *et al*., 2014; Barraclough, 2015; Nuismer & Harmon, 2015; Manceau *et al*., 2017). For mutualism, benefits to both partners favour increased interaction frequency and efficiency, establishing interspecific coalitions (Cafaro & Currie, 2005). If one or both mutualistic partners become particularly specialized in their interaction, their phylogenies could become enmeshed (e.g. Currie *et al*., 2003), as speciation of one partner precipitates co‐speciation by the other. Co‐speciation is especially likely if mutualistic interaction benefits reproduction by one or both partners, increasing reproductive isolation within partner lineages (Hembry *et al*., 2014; e.g. pollination systems: Silvieus *et al*., 2008; Althoff *et al*., 2014; van der Niet *et al*., 2014).

By contrast, negative interactions among competitors in the same or different clades favour dissociation (Mahler *et al*., 2010; Yoder & Nuismer, 2010; Barraclough, 2015; Nuismer & Harmon, 2015). Whether coexistence and dissociation are ecologically and evolutionarily feasible depends on the availability of alternative resources, the possibility of using common resources differently, and competition symmetry (Mahler *et al*., 2010; Burns & Strauss, 2011). If competition is symmetrical (e.g. equal access to limiting resources and no direct interference) and alternatives are available, adaptation by one partner that reduces interaction frequency or intensity benefits both partners and so may not induce any evolutionary response by the second partner. Thus, the phylogenies of clades of symmetrically competing species should be less congruent than those of mutualists. If, instead, competition is asymmetrical, with one partner dominating consistently, evolutionary repulsion should promote dissolution, or loss, of competitive interactions, with speciation occurring largely independently in paired lineages that include some competitors (Yoder & Nuismer, 2010).

Finally, interactions that involve host and antagonist partners, like predation, herbivory, or parasitism, promote a third set of possible evolutionary responses (Nyman, 2010; Yoder & Nuismer, 2010; Barraclough, 2015; Maron *et al*., 2019). Such interactions are generally viewed as promoting arms races, whereby evolution of new host defences favours evolution of new antagonist weapons, prompting new host defences, and so on (Janz, 2011; Marquis *et al*., 2016). According to this scenario, co‐speciation should be common in the co‐phylogenies of hosts and antagonists. However, interactions between specialized antagonists, such as parasites or insect herbivores, may foster a different pattern if hosts combat diverse antagonistic species with a battery of defences. Although interactions with individual antagonist species may select for host defences, in the context of the general battle that hosts wage against all antagonists, individual interactions may have little influence on speciation (Yoder & Nuismer, 2010). By contrast, a targeted host response to a specialized antagonist could increase the advantages of switching to an alternative host species (Silvieus *et al*., 2008), which in turn could foster antagonist speciation (Jermy, 1984; Janz, 2011; Hardy & Otto, 2014).

These considerations foster the expectation that specific types of interspecific interactions generate contrasting co‐phylogenetic patterns (Yoder & Nuismer, 2010; Hembry *et al*., 2014), although this expectation has seldom been assessed directly. Instead, co‐phylogenetic methods are generally used to examine specific interactions; for example, antagonism (Nyman, 2010), mutualism (Cruaud *et al*., 2012), and competition (Sweet *et al*., 2016). The results of such studies often support expectations (e.g. Nyman, 2010; Cruaud *et al*., 2012; Sweet *et al*., 2016; Navaud *et al*., 2018), but whether the relative frequencies of different types of macroevolutionary events (co‐speciation, association switching, association loss) differ between all three interaction types in similar conditions remains largely unexamined.

Animal pollination creates an economic system in which all these interactions play out. This system is founded on mutualistic exchange of floral resources by immobile plants for pollen dispersal by mobile animals. Plant–pollinator mutualism also creates opportunities for antagonistic animals to consume floral resources without pollinating (Pellmyr *et al*., 1996; Hargreaves *et al*., 2009; Irwin *et al*., 2010; Borges, 2015) and for antagonistic plants to attract pollinators without rewarding them (Johnson & Schiestl, 2016). Because mutualists and antagonists on the same trophic level vie for the same benefits, they can also interact competitively (Internicola *et al*., 2006; Hazlehurst & Karubian, 2018). Thus, the interactions associated with animal–pollination systems encompass mutualism, competition, and antagonism, and so should be associated with the different macroevolutionary patterns already described. However, the strength of such patterns likely varies, being more evident for specialized associations than for diffuse, generalized interactions.

**Figure 1:**
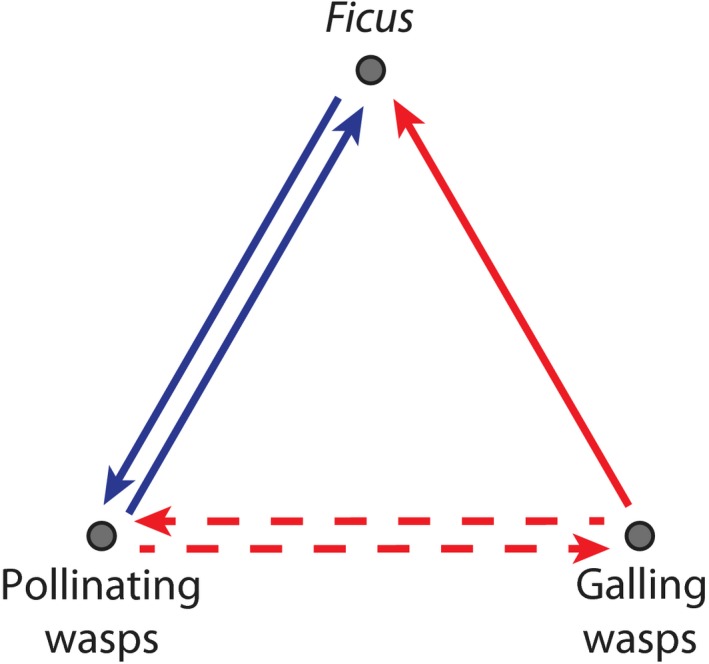
Interaction network between *Ficus* and pollinating and galling wasps, including mutualism of *Ficus* and pollinating wasps, competition between pollinating and galling wasps, and antagonism (parasitism) of *Ficus* by galling wasps. Blue and red arrows indicate positive and negative interactions, respectively. The arrows linking pollinating and galling wasps are dashed to indicate ambiguity concerning whether competition is symmetrical or asymmetrical, with one partner having a consistent advantage.

The specialized inflorescences (syconia) of the *c*. 750 species of figs (*Ficus*, Moraceae) are microcosms of interspecific interactions with a > 60 Myr macroevolutionary history (Herre *et al*., 2008; Silvieus *et al*., 2008; Compton *et al*., 2010; Segar *et al*., 2013). A syconium is an infolded receptacle that encloses 50–7000 tiny, unisexual flowers, which can be accessed directly only by a small apical pore (Verkerke, 1989). Each fig species usually depends on a single highly specialized pollinating wasp species (Hymenoptera, Chalcidoidea, Agaonidae; Herre *et al*., 2008) to disperse its pollen. In addition to pollinating, these wasps lay their eggs in the ovaries of some pollinated fig flowers, inducing gall formation by the endosperm, which the resulting larvae consume as they develop into the next adult generation (Weiblen, 2002; Jansen‐González *et al*., 2012). As pollinating wasp species generally rely on a single fig species (Cook & Segar, 2010; Yang *et al*., 2015), pairs of fig and pollinating‐wasp species engage in highly specialized mutualisms (Fig. 1). Many fig species also host a diverse group of nonpollinating (galling) wasp species (Rasplus *et al*., 1998; Weiblen, 2002) that represent several independent origins of fig parasitism (Heraty *et al*., 2013). These species belong to different chalcid families (Pteromalidae, Eurytomidae, Ormyridae, Torymidae) or subfamilies (Sycophaginae, Tetrapusiinae) than the pollinating species (Heraty *et al*., 2013). Galling species oviposit before or simultaneously with pollinating species and induce galls in the nucellus of unfertilized ovules, on which their larvae feed. Other species oviposit into developing galls, usurping gall tissue from the resident larvae (kleptoparasites), or into ovaries with maturing seeds (seed predators). These parasitic wasps rely on figs to reproduce, but provide no benefit to figs (Pereira & do Parado, 2005; Marussich & Machado, 2007) and so act as antagonists (Fig. 1). Many parasitic wasp species resemble pollinating species with respect to oviposition sites and resource demands and may mimic pollinating wasps, limiting possibilities for the evolution of effective fig defences (Bronstein, 1991). Furthermore, because both mutualistic pollinating and parasitic wasp species require the same resource for reproduction (fig ovules), they can compete with each other (West *et al*., 1996; Raja *et al*., 2015). The nature of competition likely varies depending on the fauna of parasitic wasps, their relative timing of oviposition, dependence on fertilized ovules, and so on (Segar *et al*., 2013). When pollinating and galling wasps oviposit concurrently, pollinating wasps may be superior competitors, as they are generally more abundant (Segar *et al*., 2013). Thus, all three classes of interspecific interactions can occur within a single fig syconium, providing a singular opportunity to compare the co‐phylogenetic relationships associated with mutualism, competition, and parasitism (Silvieus *et al*., 2008).

In addition to the expectations already outlined, some evidence suggests that relationships involving galling wasps may differ, depending on whether the fig species is monoecious or (functionally) dioecious. *Ficus* is ancestrally monoecious, but dioecy has evolved independently at least twice (Herre *et al*., 2008). Owing to differences between monoecious and dioecious fig species in oviposition opportunities for galling wasps, dioecious species generally support fewer galling wasp species and individuals (Kerdelhué & Rasplus, 1996a; Weiblen *et al*., 2001). Thus, pollinators of dioecious species should experience less competition with galling wasps than pollinators of monoecious species do. Consequently, interactions with the host fig species may play a stronger role in the micro‐ and macroevolution of the pollinators of dioecious figs. In keeping with this possibility, a study of relatively infrequent cases of two pollinator species using the same fig species found that all pollinator pairs were sister species for dioecious fig species, whereas this was true for only a third of cases involving monoecious fig species (Yang *et al*., 2015).

Here, we compare the co‐phylogenies of a local sample of figs and pollinating and galling wasps to assess whether their co‐diversification patterns differ, as expected from their contrasting interactions. Our sample involves 26 native fig species and their associated wasps from the tropical rainforest at Xishuangbanna, China. Using phylogenies for these species based on newly derived gene sequences, we test whether the overall co‐phylogenetic congruence and relative frequencies of inferred co‐speciation, association duplication, switching, and loss differ for the three pairs of interaction partners. The latter results are compared with the expectations we outlined earlier for macroevolutionary consequences of mutualism, competition, and antagonism. We also assess whether fig–pollinator co‐diversification differs between monoecious and dioecious fig species. By comparing the relative frequencies of different macroevolutionary events for clades that engage in different interaction types, our analysis alleviates several potential problems associated with inferring the macroevolutionary consequences of ecological interactions (see Potential study limitations section).

## Materials and Methods

### Species sampling and DNA sequencing

Phylogenetic analysis was based on gene sequences of DNA extracted from fig leaves and wasps collected in the tropical rainforest at Xishuangbanna, China (21°41′N, 101°25′E). Sampling involved 13 monoecious and 13 dioecious fig species with various growth habits that belong to five subgenera (Supporting Information Tables S1, S2). The local flora includes seven additional common fig species, but they are not included because of gene amplification failure. Leaves of one individual were collected for each fig species during November or December 2014 (Tables S1, S2) and dried with silica gel. We later extracted total genomic DNA from 20–30 mg of dried leaf material using the Plant DNA Kit DP305 (Tiangen, Beijing, China). Three nuclear genes, ITS, ETS, and *G3pdh* (glyceraldehydes 3‐phosphate dehydrogenase) and one chloroplast gene *trnL‐F* (intergenic spacer between the 3′ exon of *trnL* and *trnF*) were sequenced to reconstruct the *Ficus* phylogeny (Table S2). *Castilla elastica* (Moraceae, Castilleae) was included as an outgroup (Rønsted, 2005), using gene sequences obtained from GenBank (Table S2; Notes S1).

The 236 wasps considered in this study were collected from the same rainforest from 2008 to 2018 and belong to 28 primarily pollinating species (Agaonidae: 16 Agaoninae, 12 Kradibiinae) and 33 parasitic species (30 Pteromalidae; 3 Agaonidae, Sycophaginae: Tables S1, S3). Wasps were captured as they emerged from at least 10 figs per tree, which had been placed in individual mesh bags. These figs were collected before anthers began maturing (D‐phase; Galil & Eisikowitch, 1968; Patel, 1996). For locally abundant fig species (eight monoecious, eight dioecious; see Table S1), wasps were sampled from five or six trees, whereas for less abundant species (five monoecious and five dioecious; Table S1) wasp sampling involved three to five trees. Upon emergence, wasps were sorted to genus based on morphology. Then, 1–10 individuals were randomly collected for each species and stored in 95% ethanol at −20°C. The sample of Agaonid wasps included two species that have been identified as likely parasitic cheaters, rather than mutualistic pollinators (*Eupristina* sp. 1 from *Ficus altissima*,* Eupristina* sp. 3 from *Ficus microcarpa*; Peng *et al*., 2008; Y. Q. Peng, unpublished). These species have reduced structures for carrying pollen, and most figs entered by single females produce no seeds or galls, but many offspring wasps. We retained these species in the phylogenetic analysis to illustrate their relationships, but we excluded them from the co‐phylogenetic analyses because they are not mutualists and are distantly related to and functionally different from the other parasitic wasp species. All other parasitic wasp species produce flower galls on which their larvae feed (Kerdelhué *et al*., 2000; Compton *et al*., 2018; Y. Q. Peng, unpublished), so we refer to them as galling wasps. Unlike the pollinating wasps, the galling species lay eggs in ovules from outside syconia using elongate ovipositors (Kerdelhué *et al*., 2000). Two species from *Ficus curtipes*,* Diaziella yangi* and *Lipothymus* sp., were previously considered to be parasitic gallers (Xu *et al*., 2005; Zhang *et al*., 2008) but have since been recognized as secondary gallers that stimulate galls generated by pollinators (Chen *et al*., 2013). We treated these species as parasitic gallers in the analyses reported here, but results do not differ qualitatively if they are excluded (see Figs S1, S2). We compared whether the presence of galling wasp species differed among monoecious and dioecious *Ficus* species with Fisher's exact test.

To reconstruct the phylogenies of the pollinating and galling wasps, we extracted the entire genomic DNA from all 236 wasps and sequenced the nuclear ribosomal genes 18S rRNA (variable regions V3–V5) and 28S rRNA (D4–D5 expansion regions) and the mitochondrial cytochrome c oxidase subunit I. DNA was extracted using the E.N.Z.A.^®^ Insect DNA Kit (Omega Bio‐tek, Norcross, GA, USA). Outgroup representatives for the wasp phylogenies included four species of the superfamily Chalcidoidea (*Sycophaga*,* Ficomila*,* Megastigmus*, and *Trichogramma*) for the pollinating wasp phylogeny (Cruaud *et al*., 2012) and three species of Agaonidae (*Ceratosolen fusciceps*,* Ceratosolen gravelyi*, and *Ceratosolen emarginatus*) for the galling wasp phylogeny. Gene sequences for the outgroup species and six species of pollinating wasps were obtained from GenBank (see Table S3).

Gene sequencing for the focal fig and wasp species employed largely the same methods. Gene amplification involved procedures described by Baraket *et al*. (2010) for *trnL‐F* and by Cruaud *et al*. (2012) for all other genes. Amplification products were sequenced directly using the ABI Prism BigDye^®^ Terminator v.3.1 Ready Reaction Cycle Sequencing Kit (Applied Biosystems, Foster City, CA, USA). For some nuclear genes of some figs, direct sequencing produced polymorphic reads. In these cases, we cloned the amplification products with JM109 cells using the pGEM^®^‐T Vector System (Promega, Madison, WI, USA) and sequenced five colonies using M13 forward and reverse primers. Then we used degenerate bases to represent polymorphic base sites. All gene sequences have been deposited in GenBank (see Tables S2, S3).

### Phylogeny reconstruction

Using the gene sequences, we inferred separate phylogenies for *Ficus*, pollinating wasps, and galling wasps with common methods. Gene sequences were aligned using clustalW in bioedit v.7.2.3 (Hall, 1999). Bayesian inference was conducted with starbeast (*beast), which can infer a shared species tree from multiple gene loci and multiple individuals per species using the multispecies coalescent model (Heled & Drummond, 2010; Bouckaert *et al*., 2014). The best‐fitting substitution model for each gene was selected with jmodeltest (Darriba *et al*., 2012) using the Akaike information criterion. We used the most parameterized model available in starbeast for each partition. Markov chain Monte Carlo analyses involved 100 million generations for figs and pollinating wasps and 10 million generations for galling wasps, both of which allowed chain convergence. We regarded the first 25% of generations as burn‐in iterations, so only trees collected during subsequent generations were used to estimate the posterior probability with treeannotator (Bouckaert *et al*., 2014). Details of the phylogenetic results for *Ficus* and pollinating and galling wasps are summarized in Note S1 and Fig. S3.

### Co‐phylogenetic analyses

The extent and nature of co‐diversification of unrelated clades can be assessed based on either of two assumptions concerning the coordination of speciation, as inferred from the clade phylogenies. Contingent divergence occurs when speciation in an ‘antecedent’ clade precedes and precipitates speciation in a ‘reactive’ clade, which we denote as ‘reactive|antecedent’ (read reactive given antecedent). Thus, to assess contingent congruence, the phylogeny of the putative antecedent clade serves as the reference topology to which the phylogeny of the putative reactive clade is compared. By contrast, with independent divergence, speciation in either clade can precede speciation in the other clade, depending on circumstance. We used two approaches that incorporate these perspectives to characterize, quantify, and compare the co‐divergence of *Ficus*, pollinating wasps, and galling wasps: a Procrustean approach to infer co‐phylogenetic concordance (implemented in the R package paco 0.4.1; Balbuena *et al*., 2013), and a parsimony‐based approach for co‐phylogeny reconstruction (implemented in jane 4; Conow *et al*., 2010).

PAco assesses the overall topological similarity (congruence) of the phylogenies of two unrelated clades (*X* and *Y*), based on either the contingency or independence assumption (Balbuena *et al*., 2013; Hutchinson *et al*., 2017a). Overall congruence is measured inversely by the residual sum of squared differences between the two phylogenies ($m_{\mathrm{XY}}^{2}$, where small $m_{\mathrm{XY}}^{2}$ indicates extensive congruence). Correspondingly, the squared residual for the specific association of species *y* with species *x*, $e_{\mathrm{xy}}^{2}$, represents its contribution to $m_{\mathrm{XY}}^{2}$. The statistical significance of $m_{\mathrm{XY}}^{2}$ is assessed with a randomization test of the null expectation that taxa in the two clades associate randomly. We conducted PAco with the paco (v.0.3.2; Hutchinson *et al*., 2017a) and ape (v.5.2; Paradis *et al*., 2004) packages in R version 3.5.2 (R Core Team, 2018). Distances between species within a clade were measured based on the sum of their connecting branch lengths along the inferred phylogeny. Congruence was tested based on 1000 random permutations. To assess congruence for *Ficus* and mutualistic pollinating wasp we assumed independent divergence, reflecting their mutual dependence. By contrast, for *Ficus* and parasitic galling wasps we assumed contingent co‐divergence with galling wasps as the reactive clade, given their unilateral dependence on figs. As the nature of competition between pollinating and galling wasps is uncertain, we conducted three analyses that allowed independent divergence (symmetrical competition) or contingent co‐divergence (asymmetrical competition) with pollinators as either antecedent or reactive.

jane estimates the frequencies of different types of macroevolutionary events based on contingent co‐divergence. The events assessed by jane include the following: *co‐speciation* – speciation occurs concurrently in the reference and comparator clades; *duplication* – speciation in the comparator clade occurs without speciation in the reference clade and both new comparator species remain associated with the ancestral reference species; *association switching* – like duplication, except that one new comparator species establishes a novel association with a species on a different branch of the reference phylogeny; *association loss* – speciation occurs in only the reference clade and the existing comparator species associates with only one new reference species (or sampling error); and *failure to diverge* – speciation occurs in only the reference clade and the existing comparator species associates with both new reference species. Given the ‘known’ historical relationships (phylogenies) of extant species in the reference and comparator clades and their known contemporary associations, inference of the historical co‐phylogenetic relationships involves two steps (Conow *et al*., 2010). During the first step, a genetic algorithm identifies which ‘population’ of possible relative timings of internal nodes for the two phylogenies is most ‘fit’. During the second step, the putative registered chronology and dynamic programming are used to infer the set of macroevolutionary events that generated the observed contemporary associations. Given the inferred set of macroevolutionary events for a specific timing, its fit equals the sum over all events of the inferred number of transitions weighted by their individual ‘cost’. jane then uses the best‐fitting (i.e. lowest total cost) timing as the basis for constructing a new population of possible timings, and this two‐step process is repeated for the number of iterations (‘generations’) specified by the user.

Our implementation of jane largely followed the parameters used by Cruaud *et al*. (2012) in their analysis of the co‐phylogenies of *Ficus* and pollinating wasps for a global sample of species. Specifically, we set the costs per event as 0 for co‐speciation and 1 for duplication, branch switching, loss, and divergence failure, which is the primary cost structure considered by Cruaud *et al*. All analyses ran for 40 generations and considered 1000 populations, except that the analyses of *Ficus* and pollinating wasps involved 4000 populations.

Being based on contingent co‐divergence, jane infers macroevolutionary events by comparing the phylogeny of a comparator (putative reactive) clade with that of a reference (putative antecedent) clade (i.e. comparator|reference) (Conow *et al*., 2010). This approach is appropriate for the parasitic relation of galling wasps with *Ficus*, as the evolution of galling wasps reasonably follows that of *Ficus*, to the extent that their histories are linked. By contrast, the choice of reference and comparator clades is not obvious for the mutualistic association of *Ficus* and pollinating wasps, or possibly for pollinating and galling wasps, depending on the nature of their competitive interactions. For these cases, the inferred timings and types of co‐phylogenetic events could depend on the choice of reference and comparator clades, possibly leading to spurious conclusions. To address this problem, we conducted two jane analyses for each pair of clades. Comparison of the two analyses for *Ficus* and galling wasps is informative, because the distribution of events for gallers|*Ficus* should be more indicative of the actual history than that for *Ficus*|gallers. This information can then be used to identify whether the choice of reference and comparator clades affects the outcome for the other two analyses and, if so, which choice is preferable.

We compared the inferred relative frequencies of the five classes of co‐phylogenetic events for the associations of pollinating (excluding cheaters) and galling wasps with *Ficus*,* Ficus* and galling wasps with pollinating wasps, *Ficus* and pollinating wasps with galling wasps, and monoecious and dioecious *Ficus* with pollinating wasps. For most co‐phylogenetic analyses, jane identified several (four or fewer) different event distributions with the same minimal cost. For example, the analysis of the associations of *Ficus* and pollinating wasps found two lowest‐cost sets of distributions for co‐speciation, duplication, switching, loss, and failure to diverge (9, 2, 16, 0, 0; 10, 2, 15, 1, 0), representing 79.5% and 20.5% of all solutions, respectively. In these cases, we calculated the average probability of rejecting the null hypothesis of identical event frequencies using the event distributions for all outcome sets, weighted by their respective relative frequencies.

## Results

**Figure 2:**
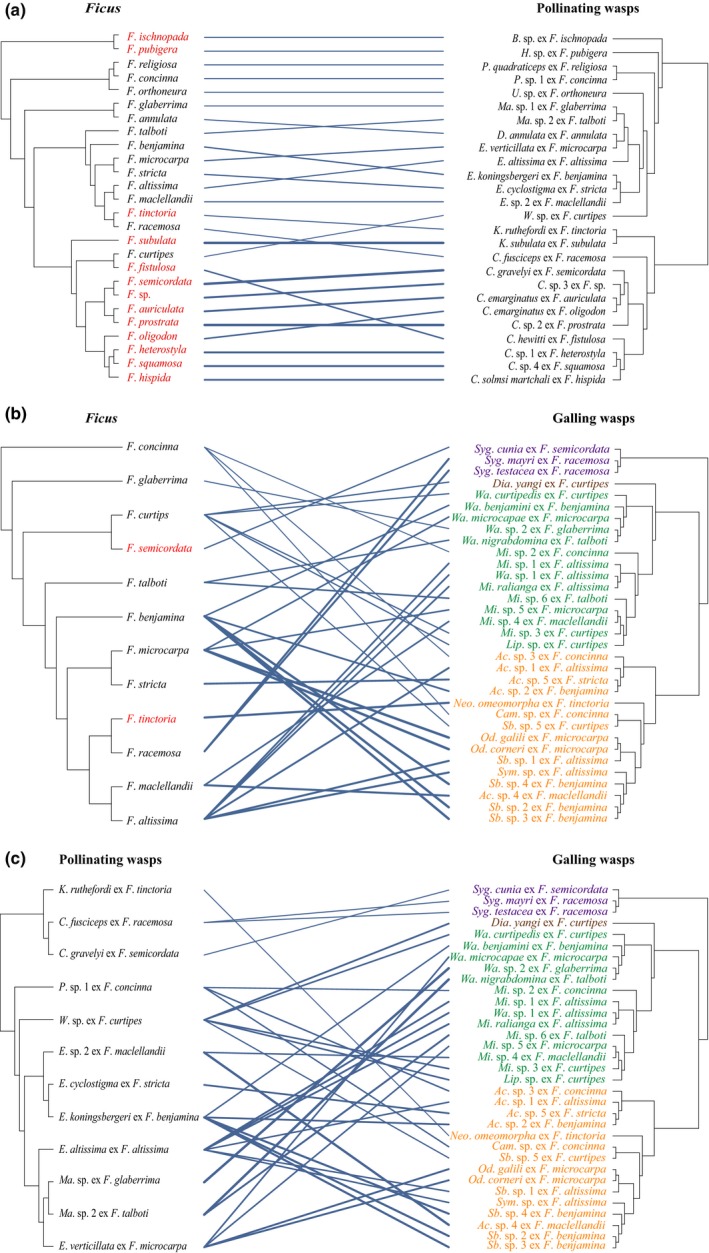
Co‐phylogenetic patterns between (a) *Ficus* and pollinating wasps, (b) *Ficus* and galling wasps, and (c) pollinating and galling wasps. For *Ficus*, red and black labelling distinguishes dioecious and monoecious species, respectively. Subfamilies Epichrysomallinae, Otitesellinae, Sycophaginae, and Sycoecinae are shown in orange, green, purple, and brown in (b) and (c). Solid lines connecting species in the left‐ and right‐hand phylogenetic trees indicate observed associations. Line thickness varies positively with the contribution of a particular association to overall phylogenetic congruence. The ‘ex’ labels identify the host fig species of individual wasp species. Abbreviations in (a), (b) and (c) for figs: *F*., *Ficus*. Abbreviations in (a) and (b) for fig pollinating wasps: *B*., *Blastophaga*;* C*., *Ceratosolen*;* D*., *Deliagaon*;* E*., *Eupristina*;* H*., *Hederagaon*;* K*., *Kradibia*;* P*., *Platyscapa*;* U*., *Umagaon*;* W*., *Waterstoniella*. Abbreviations in (b) and (c) for galling wasps: *Ac*., *Acophila*;* Cam*., *Camarothorax*;* Dia*., *Diaziella*;* Lip*., *Lipothymus*;* Mi*., *Micranisa*;* Neo*., *Neosycophila*;* Od*., *Odontofroggatia*;* Sb*., *Sycobia*;* Syg*., *Sycophaga*;* Sym*., *Sycophilomorpha*;* Wa*., *Walkerella*.

The pairwise associations between *Ficus* and pollinating and galling wasps differed considerably. Each pollinating wasp species associated with only one *Ficus* species (Fig. 2a). By contrast, the 33 species of galling wasps associated with only 12 *Ficus* species. Galling wasps associated nonrandomly with fig species, as 10 of the 12 host species are monoecious (Fig. 2b; Fisher's exact test, *P* < 0.01). Eight *Ficus* species (all monoecious) hosted multiple (up to six) species of galling wasps; however, each galling wasp species used syconia of only one *Ficus* species and correspondingly associated with only one species of pollinating wasp (Fig. 2c). The multiple galling wasp species associated with individual species of *Ficus* and pollinating wasps generally represented different subfamilies (Fig. 2b,c).

The extent of historical co‐diversification between figs and pollinating and galling wasps, as quantified by the distance‐based PAco analyses, differs depending on the nature of their interactions. The overall analysis for figs and their pollinating wasps identified significant phylogenetic congruence ($m_{\mathrm{XY}}^{2}$ = 0.487, *P *<* *0.001). However, this result obscures heterogeneity, as illustrated by the different thicknesses of the association lines in Fig. 2a. Specifically, the diversification histories of figs and pollinating wasps were significantly congruent for dioecious figs ($m_{\mathrm{XY}}^{2}$ = 0.232, *P* < 0.001) but not for monoecious figs ($m_{\mathrm{XY}}^{2}$ = 0.591, *P *>* *0.3). Similarly, the phylogenetic history of galling wasps was not congruent with that of their fig hosts ($m_{\mathrm{XY}}^{2}$ = 1.399, *P *>* *0.3; Fig. 2b), most of which are monoecious. By contrast, pollinating and galling wasps hosted by these fig species exhibited significant phylogenetic congruence (Fig. 2c), regardless of whether the analysis considered independent divergence ($m_{\mathrm{XY}}^{2}$ = 0.670, *P *<* *0.001) or contingent divergence (Gallers|Pollinators, $m_{\mathrm{XY}}^{2}$ = 0.00008, *P *<* *0.001; Pollinators|Gallers, $m_{\mathrm{XY}}^{2}$ = 0.00006, *P *<* *0.001).

**Figure 3:**
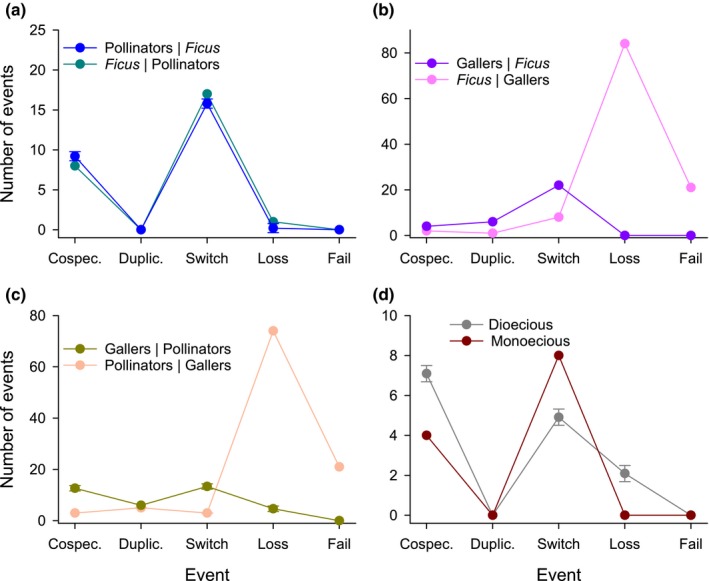
Comparisons of the symmetry of distributions of co‐phylogenetic events (co‐speciation, duplication, association switch, association loss, failure to diverge) inferred by jane for (a) *Ficus* and pollinating wasps, (b) *Ficus* and galling wasps, and (c) pollinating and galling wasps, and (d) of the associations of pollinating wasps with monoecious and dioecious *Ficus* species. In all cases *X*|*Y* denotes that clade *Y* was the reference clade and clade *X* was the comparator clade. In (a), (c) and (d), error bars indicate ± SD for cases in which jane identified several different sets of outcomes associated with the same minimal cost.

Comparisons of jane analyses that differed with respect to which clade served as reference and comparator groups revealed informative differences between the pairwise contrasts for *Ficus* and pollinating and galling wasps (Fig. 3). For the mutualistic relation of *Ficus* and pollinating wasps, the results differed little if *Ficus* served as the reference or comparator clade (Fig. 3a). As Pollinators|*Ficus* fit slightly better than *Ficus*|Pollinators (total cost equal to 16 vs 18, respectively; Fig. 3a), we use the Pollinators|*Ficus* results in subsequent comparisons between interaction types. For this association, the relative frequencies of macroevolutionary events did not differ significantly between monoecious and dioecious fig species (*P* < 0.2; Fig. 3d). For the co‐phylogenetic associations of *Ficus* and their parasitic galling wasps, the analysis that reflected their ecological interaction, Gallers|*Ficus*, fit much more parsimoniously than the unrealistic *Ficus*|Gallers option (total cost equal to 28 and 114, respectively). These results differed, in that jane inferred more host switching but much less association loss and divergence failure for the realistic relation (Fig. 3b). A similar contrast was evident for the association of pollinating and galling wasps (Fig. 3c). In this case, Gallers|Pollinators fit much more parsimoniously (total cost equal to 24 and 103, respectively). Consequently, we use the results for Gallers|*Ficus* and Gallers|Pollinators in subsequent comparisons between interaction types.

**Figure 4:**
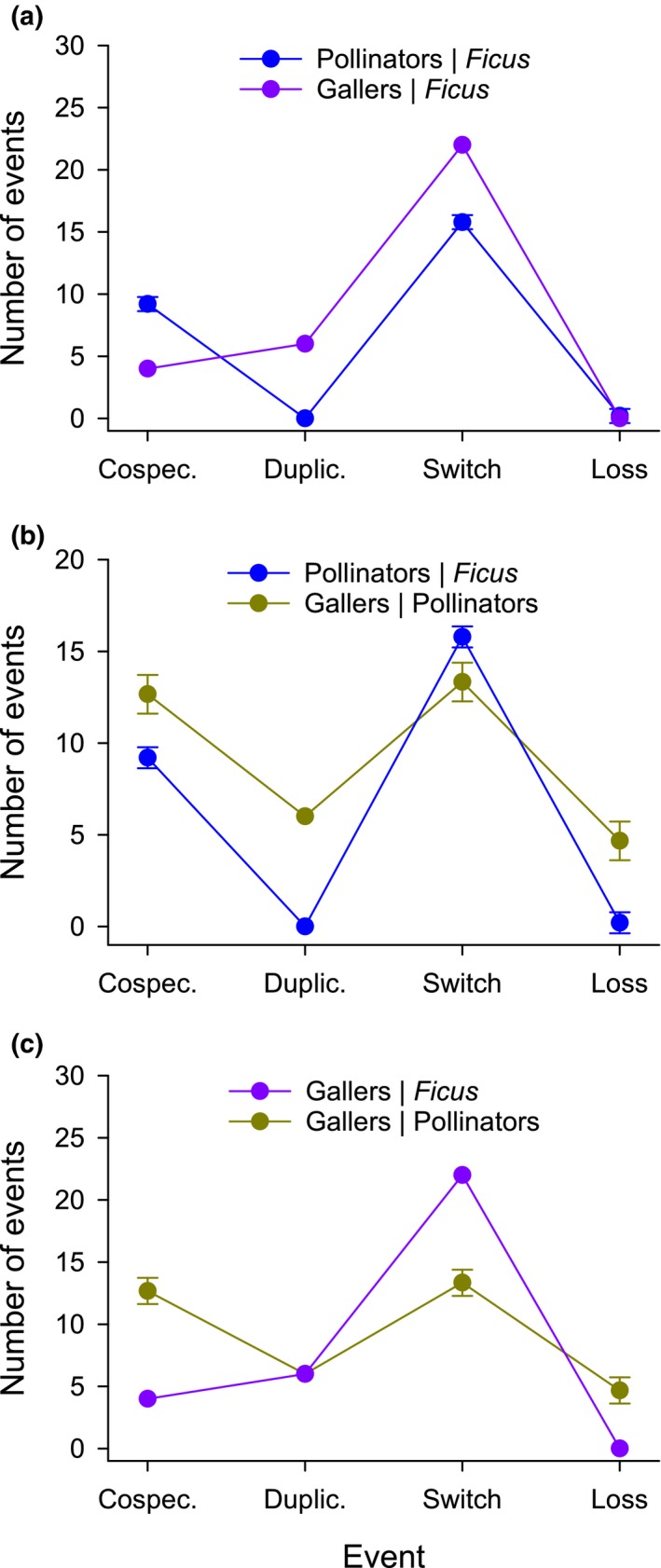
The frequencies of different types of co‐phylogenetic events (co‐speciation, duplication, association switch, association loss) inferred by jane for pairwise comparisons of the phylogenies of (a) pollinating wasps and galling wasps in the context of *Ficus* evolution, (b) *Ficus* and galling wasps in the context of pollinating wasp evolution, and (c) galling wasps in the context of *Ficus* and pollinating wasp evolution. *X*|*Y* denotes that clade *Y* was the reference clade and clade *X* was the comparator clade; error bars indicate ± SD for cases in which jane identified several different sets of outcomes associated with the same minimal cost. Lines linking events are provided to aid comparison of distributions for different taxa but convey no quantitative information.

The co‐diversification characteristics inferred by the jane analyses reveal differences in the macroevolutionary processes that generated the observed overall patterns of phylogenetic congruence (Fig. 4). The distributions of event frequencies differed significantly for co‐diversification of mutualistic pollinators and parasitic gallers with *Ficus* (averaged Fisher's exact tests, *P *<* *0.025). Specifically, compared with galling wasps, co‐diversification of pollinating wasps involved more co‐speciation and less duplication and host switching (Fig. 4a). The relative event frequencies for mutualists (Pollinators|*Ficus*) and competitors (Gallers|Pollinators) also differed significantly (*P *<* *0.05). In this case, co‐diversification of competitors involved more co‐speciation, association duplication, and loss than that of mutualists (Fig. 4b). Finally, the significant difference in event distributions for galling wasps as antagonists of *Ficus* and competitors of pollinating wasps (*P *<* *0.025) involved more co‐speciation and association loss, but less association switching by competitors than by antagonists (Fig. 4c). In addition to these patterns, note the moderate inferred frequency of association losses involving pollinating and galling wasps compared with that of either co‐phylogeny involving *Ficus* species (Fig. 4b,c).

## Discussion

### Interaction type and co‐diversification

The co‐diversification patterns detected for the phylogenies of a local assemblage of fig species and their pollinating and galling wasps largely support expectations for mutualists and antagonists, but they are less consistent for competitors. The co‐phylogeny of *Ficus* and their pollinating wasps reflects their mutualistic interdependence. As expected, this co‐phylogeny exhibited significant congruence, although only for interactions involving dioecious fig species (Fig. 2a; see Interaction of interactions section). The inferred relative frequencies of macroevolutionary events differed little if *Ficus* or pollinators served as the reference clade in the jane analysis (Fig. 3a), indicating reciprocal diversification. Finally, as expected, the histories of the mutualists involved more co‐speciation than the host–antagonist (*Ficus*–galler) co‐phylogeny (Fig. 4a). Relatively high incidence of co‐speciation is a common finding of macroevolutionary studies of figs and their pollinating wasps, whether considering samples from single sites, biogeographic regions, or the global biota (Jousselin *et al*., 2008; Silvieus *et al*., 2008; Cruaud *et al*., 2012). Phylogenetic congruence has also been observed for more generalized pollination systems (Hutchinson *et al*., 2017b), and co‐speciation is similarly prevalent in the co‐diversification of other specialized mutualistic and commensal associations (e.g. Currie *et al*., 2003; Hoyal Cuthill & Charleston, 2012; Chen *et al*., 2017).

The phylogenetic association of *Ficus* hosts with parasitic galling wasps differed from that with pollinating wasps, reflecting the antagonistic nature of their interaction (also see Weiblen & Bush, 2002; Silvieus *et al*., 2008). Each *Ficus* species hosted a unique set of galling wasps, usually comprised of distantly related species from different subfamilies (Fig. 2b). Consequently, the jane analysis that incorrectly specified the history of galling wasps as the reference (antecedent) phylogeny inferred association loss and divergence failure as the most common macroevolutionary events (Fig. 3b). Finally, compared with the mutualist co‐phylogeny, the host–antagonist history involved more host switching (Fig. 4a; also see Weiblen & Bush, 2002), generating the taxonomic diversity of galling species associated with individual pollinating wasp species. This difference arose even though galling wasps interacted with only 12 of the 26 *Ficus* species studied and their pollinating wasps. The prevalence host switching could reflect two nonexclusive influences. It should arise if hosts respond to diverse sets of specialized antagonists with batteries of defences (Jermy, 1984; Janz, 2011; Hardy & Otto, 2014), and is commonly observed for figs and galling wasps (Cook & Segar, 2010) and other antagonistic associations (Doña *et al*., 2017; Hsu *et al*., 2018; Purcell *et al*., 2018). Host switching could also be promoted by competition among multiple galling species contesting for access to ovules of the same fig species (e.g. Kerdelhué & Rasplus, 1996b). The relative importance of these influences on co‐diversification of figs and their parasites awaits focused analysis.

Compared with mutualism and antagonism, the results concerning competitive associations between pollinating and galling wasps align less consistently with expectations. The phylogenetic histories of the competitor clades were significantly congruent, regardless of the nature of the assumed competition (Fig. 2c; also see Marussich & Machado, 2007). However, alternative scenarios for the nature of competition support very different inferences concerning the competitive influences of pollinating and parasitic wasps on each other (Fig. 3c). If galler speciation is assumed to precede pollinator divergence (Pollinators|Gallers), jane analysis inferred very high frequencies of association loss and divergence failure. These events both involve independent diversification but differ in whether individual species in the reactive clade continued associating with one (association loss) or both (divergence failure) derived species in the antecedent clade. By contrast, for the Gallers|Pollinators scenario, such macroevolutionary independence was inferred to have occurred rarely, whereas co‐diversification events (co‐speciation and association switching) were relatively more common. That this scenario supported a much more parsimonious inference suggests that diversification of galling wasps primarily reacted to that of pollinating wasps. Based on this conclusion, the inferred high relative frequency of association switching, compared with mutualists or antagonists (Fig. 4b,c), is consistent with the expected macroevolutionary consequences of interspecific competition. By contrast, the corresponding high inferred frequencies of co‐speciation compared with mutualistic and host–parasite associations (Fig. 4b,c) and of duplication compared with mutualists (Fig. 4b) contradict the expectation of evolutionary dissociation of competitors. This contrast likely reflects heterogeneous competition (Sweet *et al*., 2016) associated with the diverse biology of co‐occurring parasitic wasps (see Borges, 2015). In particular, galling wasp species likely experience limited or intense competition with pollinating species, or even with other cohabiting galling species, depending on whether they oviposit before or simultaneously or after each other. Thus, just as competition has manifold ecological consequences (Aschehoug *et al*., 2016), its macroevolutionary consequences may be more diverse than those of mutualism and parasitism (Drury *et al*., 2016). This complexity may partially contribute to the few published studies of co‐diversification involving clades of competitors (but see Sweet *et al*., 2016).

### Interaction of interactions

In contrast to the pairwise comparisons between mutualism, antagonism, and competition that are the focus of our analysis, all three interaction types occur simultaneously in fig microcosms and other communities (e.g. Cafaro & Currie, 2005). This complexity creates the possibility that the co‐diversification characteristics of one interaction type, such as mutualism, might differ depending on the nature of the other interactions in which species engage. Such interference could be identified by comparison of macroevolution of two interaction types in the presence or absence of a third type.

Monoecious and dioecious fig species provide an opportunity for such a comparison, because galling wasps, and hence their roles as antagonists and competitors, are largely absent from dioecious species (Kerdelhué & Rasplus, 1996a; Wu *et al*., 2013). Specifically, galling wasps were absent from 11 of the 13 dioecious *Ficus* species that we studied, and the other two species interacted with only one galling species (Fig. 2). The PAco analyses detected largely isomorphic phylogenies for dioecious figs and their pollinators, as expected for shared histories (Fig. 2a). By contrast, the correspondence of the branching patterns of monoecious figs and their pollinators did not differ significantly from random expectation. In parallel with this contrast, inferred co‐diversification patterns with pollinating wasps involved somewhat more co‐speciation for dioecious figs but more host switching for monoecious figs (Fig. 3d), although this difference was not statistically significant, perhaps because of limited statistical power (13 associations for each sexual system). Similarly, Yang *et al*. (2015) found more prevalent co‐speciation and duplication but less frequent host switching for dioecious figs than for monoecious species.

The somewhat different co‐diversification patterns associated with the mutualism of pollinating wasps with monoecious and dioecious figs could reflect various evolutionary effects of interaction with galling wasps. Evolution of (mostly monoecious) figs and pollinators in the presence of gallers likely involves compromise between promoting mutualistic interactions and defending against the effects of parasitism and competition. By contrast, evolution of fig and wasp traits that promote mutualism should be less constrained in (mostly dioecious) lineages that interact weakly, if at all, with galling wasps. The resulting enhanced specialization of traits promoting fig–pollinator mutualism should correspondingly increase the likelihood of co‐speciation and decrease the opportunity for association switching. More generally, this interpretation identifies that, although specific types of ecological interaction are expected to generate particular patterns of co‐diversification, these patterns may not be fully realized when species also engage in other interactions.

### Potential study limitations

Several features of our study might constrain the scope of interpretation. The first feature involves whether the observed co‐phylogenetic patterns support inference regarding the underlying ecological and speciation processes. For example, several processes could cause the co‐phylogenetic conservatism evident in tendency of related species of pollinating wasps to occupy related *Ficus* species (Fig. 2a). One possibility involves co‐speciation, whereby interaction between partners promotes reproductive isolation for both species. Another possibility involves independent, coincident local adaptation after the partners occupy a new environment following dispersal or vicariance (Althoff *et al*., 2014; Hembry *et al*., 2014; Maron *et al*., 2019). However, this alternative seems unlikely for figs and pollinating wasps because, except for a brief dispersal period, pollinating wasps live their lives within figs. Consequently, local adaptation by pollinating wasps should arise largely from selection imposed by syconium characteristics, rather than by external environmental conditions. Similarly, rather than reflecting competitive interactions, co‐diversification of galling and pollinating wasps could simply reflect their joint association with speciating fig species. However, this possibility is not supported by the contrasting relative frequencies of different macroevolutionary events involving these clades with *Ficus* (Fig. 4a) and galling wasps with *Ficus* or pollinating wasps (Fig. 4c). Thus, biological evidence and contrasting inferred distributions of macroevolutionary events argue in favour of distinctive influences of mutualism, competition, and antagonism on the co‐diversification of figs and pollinating and galling wasps.

The second possible limitation involves our analysis of a local fig–wasp assemblage. The sampled species are a subset of the diversity of *Ficus* and associated wasps, even in tropical China (Yang *et al*., 2004), and so incompletely represent the phylogenetic relationships within clades and interactions between clades. Such incomplete sampling necessarily increases the apparent incongruence between the phylogenies of interacting clades, predisposing inference of host switching over co‐speciation (Cruaud *et al*., 2012). However, our analysis focuses on the relative, rather than absolute, frequencies of different events, particularly whether they differ depending on the functional roles played by the wasp species (e.g. was co‐speciation more common for mutualists than for hosts and antagonists?). As most pollinating and galling wasp species associate with a single *Ficus* species (Cook & Segar, 2010; Fig. 2), these relative frequencies should be more robust to incomplete sampling. The possible exception involves the comparison of co‐phylogenetic events for pollinating wasps with monoecious and dioecious *Ficus*. Thirteen species represented each sexual system, which could limit statistical power to detect different relative frequencies. Nevertheless, our analysis did identify such differences, suggesting that the sample was adequate for its intended purpose. Thus, the tripartite nature of our analysis and specific features of the fig microcosm likely mitigate the effects of sampling a local assemblage.

### Conclusions

The results of this study generally support the proposal that mutualism, competition, and antagonism can influence co‐diversification of interacting clades. Just as beneficial interactions between individuals promote coalitions and negative interactions promote dissociation, mutualism tends to facilitate co‐speciation, competition promotes interaction loss, and antagonism encourages association switching. However, the co‐phylogenetic patterns observed for figs and their associated pollinating and galling wasps do not adhere strictly to these expectations, likely for two reasons. Heterogeneous features of a particular type of interaction, such as competition, with several other species can foster diverse macroevolutionary outcomes. In addition, three‐way interactions between mutualists, competitors, and antagonists may alter the conditions that enable co‐speciation and association switching and loss. Owing to such context dependence, the expected influences of ecological interaction on co‐diversification may be most apparent for highly specific associations between evolving clades, such as exist in fig microcosms.

## Author contributions

W‐JL, Y‐QP and D‐YZ designed the research. A‐YW, Y‐QP, D‐RY, J‐FH and W‐JL conducted field sampling and identified fig and wasp species. A‐YW performed the molecular analyses and phylogenetic reconstruction, and A‐YW and LDH conducted the data analyses. A‐YW, LDH, D‐YZ and W‐JL wrote the manuscript. A‐YW and Y‐QP contributed equally to this work.

## Supporting information

Please note: Wiley Blackwell are not responsible for the content or functionality of any Supporting Information supplied by the authors. Any queries (other than missing material) should be directed to the *New Phytologist* Central Office.

**Fig. S1** Co‐phylogenetic associations of *Ficus* and galling wasps, and pollinating and galling wasp species, excluding secondary galling species.
**Fig. S2** The frequencies of different types of phylogenetic events inferred by Jane for pairwise comparisons of the phylogenies of *Ficus*, pollinating wasps and galling wasps, excluding secondary galling species.
**Fig. S3** Phylogenetic trees for *Ficus*, pollinating wasps and galling wasps.
**Notes S1** Summary of phylogenetic results for *Ficus*, pollinating and galling wasps.
**Table S1** *Ficus* and fig wasp species included in the phylogenetic and co‐phylogenetic analyses.
**Table S2** Taxonomic affinity, sexual systems and GenBank accession numbers of *Ficus* species involved in this study.
**Table S3** Ecological associations and roles, collection information and GenBank accession numbers for wasps included in this study.

## References

[nph16176-bib-0001] Althoff DM , Segraves KA , Johnson MT . 2014 Testing for coevolutionary diversification: linking pattern with process. Trends in Ecology & Evolution 29: 82–89. 24314843 10.1016/j.tree.2013.11.003

[nph16176-bib-0002] Aschehoug ET , Brooker R , Atwater DZ , Maron JL , Callaway RM . 2016 The mechanisms and consequences of interspecific competition among plants. Annual Review of Ecology, Evolution, and Systematics 47: 263–281.

[nph16176-bib-0003] Balbuena JA , Miguez‐Lozano R , Blasco‐Costa I . 2013 PAco: a novel Procrustes application to cophylogenetic analysis. PLoS ONE 8: e61048. 23580325 10.1371/journal.pone.0061048 PMC3620278

[nph16176-bib-0004] Baraket G , Abdelkrim AB , Saddoud O , Chatti K , Mars M , Trifi M , Salhi‐Hannachi A . 2010 Molecular polymorphism of cytoplasmic DNA in *Ficus carica* L.: insights from non‐coding regions of chloroplast DNA. Scientia Horticulturae 125: 512–517.

[nph16176-bib-0005] Barraclough TG . 2015 How do species interactions affect evolutionary dynamics across whole communities? Annual Review of Ecology, Evolution, and Systematics 46: 25–48.

[nph16176-bib-0006] Borges RM . 2015 How to be a fig wasp parasite on the fig–fig wasp mutualism. Current Opinion in Insect Science 8: 34–40. 10.1016/j.cois.2015.01.011 32846670

[nph16176-bib-0007] Bouckaert R , Heled J , Kuhnert D , Vaughan T , Wu CH , Xie D , Suchard MA , Rambaut A , Drummond AJ . 2014 beast 2: a software platform for Bayesian evolutionary analysis. PLoS Computational Biology 10: e1003537. 24722319 10.1371/journal.pcbi.1003537 PMC3985171

[nph16176-bib-0008] Bronstein JL . 1991 The nonpollinating wasp fauna of *Ficus pertusa*: exploitation of a mutualism? Oikos 61: 175–186.

[nph16176-bib-0009] Burns JH , Strauss SY . 2011 More closely related species are more ecologically similar in an experimental test. Proceedings of the National Academy of Sciences, USA 108: 5302–5307. 10.1073/pnas.1013003108 PMC3069184 21402914

[nph16176-bib-0010] Cafaro MJ , Currie CR . 2005 Phylogenetic analysis of mutualistic filamentous bacteria associated with fungus‐growing ants. Canadian Journal of Microbiology 51: 441–446. 16121221 10.1139/w05-023

[nph16176-bib-0011] Chen R , Wang Z , Chen J , Jiang LY , Qiao GX . 2017 Insect–bacteria parallel evolution in multiple‐co‐obligate‐aphid association: a case in Lachninae (Hemiptera: Aphididae). Scientific Reports 7: e10204. 10.1038/s41598-017-10761-9 PMC5579299 28860659

[nph16176-bib-0012] Chen HH , Yang DR , Gu D , Compton SG , Peng YQ . 2013 Secondary galling: a novel feeding strategy among ‘non‐pollinating’ fig wasps from *Ficus curtipes* . Ecological Entomology 38: 381–389.

[nph16176-bib-0013] Compton SG , Ball AD , Collinson ME , Hayes P , Rasnitsyn AP , Ross AJ . 2010 Ancient fig wasps indicate at least 34 Myr of stasis in their mutualism with fig trees. Biology Letters 6: 838–842. 20554563 10.1098/rsbl.2010.0389 PMC3001375

[nph16176-bib-0014] Compton SG , Chen XY , Chen Y , Hatcher MJ , Peng YQ , Quinnell RJ , Rodriguez LJ , Yu H , Ouyang A , Wei FL *et al* 2018 Host–parasitoid relationships within figs of an invasive fig tree: a fig wasp community structured by gall size. Insect Conservation and Diversity 11: 341–351.

[nph16176-bib-0015] Conow C , Fielder D , Ovadia Y , Libeskind‐Hadas R . 2010 jane: a new tool for the cophylogeny reconstruction problem. Algorithms for Molecular Biology 5: e16. 10.1186/1748-7188-5-16 PMC2830923 20181081

[nph16176-bib-0016] Cook JM , Segar ST . 2010 Speciation in fig wasps. Ecological Entomology 35: 54–66.

[nph16176-bib-0017] Cruaud A , Ronsted N , Chantarasuwan B , Chou LS , Clement WL , Couloux A , Cousins B , Genson G , Harrison RD , Hanson PE *et al* 2012 An extreme case of plant–insect codiversification: figs and fig‐pollinating wasps. Systematic Biology 61: 1029–1047. 22848088 10.1093/sysbio/sys068 PMC3478567

[nph16176-bib-0018] Currie CR , Wong B , Stuart AE , Schultz TR , Rehner SA , Mueller UG , Sung G‐H , Spatafora JW , Straus NA . 2003 Ancient tripartite coevolution in the attine ant–microbe symbiosis. Science 299: 386–388. 12532015 10.1126/science.1078155

[nph16176-bib-0019] Darriba D , Taboada GL , Doallo R , Posada D . 2012 jmodeltest 2: more models, new heuristics and parallel computing. Nature Methods 9: e772. 10.1038/nmeth.2109 PMC4594756 22847109

[nph16176-bib-0020] Doña J , Sweet AD , Johnson KP , Serrano D , Mironov S , Jovani R . 2017 Cophylogenetic analyses reveal extensive host‐shift speciation in a highly specialized and host‐specific symbiont system. Molecular Phylogenetics and Evolution 115: 190–196. 28811260 10.1016/j.ympev.2017.08.005

[nph16176-bib-0021] Drury J , Clavel J , Manceau M , Morlon H . 2016 Estimating the effect of competition on trait evolution using maximum likelihood inference. Systematic Biology 65: 700–710. 26966005 10.1093/sysbio/syw020

[nph16176-bib-0022] Galil J , Eisikowitch D . 1968 Flowering cycles and fruit types of *Ficus sycomorus* in Israel. New Phytologist 67: 745–758.

[nph16176-bib-0023] Hall TA . 1999 bioedit: a user‐friendly biological sequence alignment editor and analysis program for Windows 95/98/NT. Nucleic Acids Symposium Series 41: 95–98.

[nph16176-bib-0024] Hardy NB , Otto SP . 2014 Specialization and generalization in the diversification of phytophagous insects: tests of the musical chairs and oscillation hypotheses. Proceedings of the Royal Society of London. Series B: Biological Sciences 281: e20132960. 10.1098/rspb.2013.2960 PMC4213601 25274368

[nph16176-bib-0025] Hargreaves AL , Harder LD , Johnson SD . 2009 Consumptive emasculation: the ecological and evolutionary consequences of pollen theft. Biological Reviews 84: 259–276. 19382932 10.1111/j.1469-185X.2008.00074.x

[nph16176-bib-0026] Hazlehurst JA , Karubian JO . 2018 Impacts of nectar robbing on the foraging ecology of a territorial hummingbird. Behavioural Processes 149: 27–34. 29369784 10.1016/j.beproc.2018.01.001

[nph16176-bib-0027] Heled J , Drummond AJ . 2010 Bayesian inference of species trees from multilocus data. Molecular Biology and Evolution 27: 570–580. 19906793 10.1093/molbev/msp274 PMC2822290

[nph16176-bib-0028] Hembry DH , Yoder JB , Goodman KR . 2014 Coevolution and the diversification of life. American Naturalist 184: 425–438. 10.1086/677928 25226178

[nph16176-bib-0029] Heraty JM , Burks RA , Cruaud A , Gibson GAP , Liljeblad J , Munro J , Rasplus JY , Delvare G , Jansta P , Gumovsky A *et al* 2013 A phylogenetic analysis of the megadiverse Chalcidoidea (Hymenoptera). Cladistics 29: 466–542. 10.1111/cla.12006 34798768

[nph16176-bib-0030] Herre EA , Jandér KC , Machado CA . 2008 Evolutionary ecology of figs and their associates: recent progress and outstanding puzzles. Annual Review of Ecology Evolution and Systematics 39: 439–458.

[nph16176-bib-0031] Hoyal Cuthill JH , Charleston M . 2012 Phylogenetic codivergence supports coevolution of mimetic *Heliconius* butterflies. PLoS ONE 7: e36464. 22586474 10.1371/journal.pone.0036464 PMC3346731

[nph16176-bib-0032] Hsu YH , Cocroft RB , Snyder RL , Lin CP . 2018 You stay, but I hop: host shifting near and far co‐dominated the evolution of *Enchenopa* treehoppers. Ecology and Evolution 8: 1954–1965. 29468015 10.1002/ece3.3815 PMC5817127

[nph16176-bib-0033] Hutchinson MC , Cagua EF , Balbuena JA , Stouffer DB , Poisot T , Fitzjohn R . 2017a paco: implementing Procrustean approach to cophylogeny in R. Methods in Ecology and Evolution 8: 932–940.

[nph16176-bib-0034] Hutchinson MC , Cagua EF , Stouffer DB . 2017b Cophylogenetic signal is detectable in pollination interactions across ecological scales. Ecology 98: 2640–2652. 28734071 10.1002/ecy.1955

[nph16176-bib-0035] Internicola AI , Juillet N , Smithson A , Gigord LDB . 2006 Experimental investigation of the effect of spatial aggregation on reproductive success in a rewardless orchid. Oecologia 150: 435–441. 16941182 10.1007/s00442-006-0530-0

[nph16176-bib-0036] Irwin RE , Bronstein JL , Manson JS , Richardson L . 2010 Nectar robbing: ecological and evolutionary perspectives. Annual Review of Ecology, Evolution, and Systematics 41: 271–292.

[nph16176-bib-0037] Jansen‐González S , Teixeira SP , Pereira RAS . 2012 Mutualism from the inside: coordinated development of plant and insect in an active pollinating fig wasp. Arthropod–Plant Interactions 6: 601–609.

[nph16176-bib-0038] Janz N . 2011 Ehrlich and Raven revisited: mechanisms underlying codiversification of plants and enemies. Annual Review of Ecology, Evolution, and Systematics 42: 71–89.

[nph16176-bib-0039] Jermy T . 1984 Evolution of insect/host plant relationships. American Naturalist 124: 609–630.

[nph16176-bib-0040] Johnson SD , Schiestl FP . 2016 Floral mimicry. Oxford, UK: Oxford University Press.

[nph16176-bib-0041] Jousselin E , van Noort S , Berry V , Rasplus J‐Y , Ronsted N , Erasmus JC , Greeff JM . 2008 One fig to bind them all: host conservatism in a fig wasp community unraveled by cospeciation analyses among pollinating and nonpollinating fig wasps. Evolution 62: 1777–1797. 18419750 10.1111/j.1558-5646.2008.00406.x

[nph16176-bib-0042] Kerdelhué C , Rasplus J‐Y . 1996a The evolution of dioecy among *Ficus* an alternative hypothesis involving non‐pollinating fig wasp pressure on the fig–pollinator mutualism. Oikos 77: 163–166.

[nph16176-bib-0043] Kerdelhué C , Rasplus J‐Y . 1996b Non‐pollinating Afrotropical fig wasps affect the fig–pollinator mutualism in *Ficus* within the subgenus *Sycomorus* . Oikos 75: 3–14.

[nph16176-bib-0044] Kerdelhué C , Rossi J‐P , Rasplus J‐Y . 2000 Comparative community ecology studies on Old World figs and fig wasps. Ecology 81: 2832–2849.

[nph16176-bib-0045] Mahler DL , Revell LJ , Glor RE , Losos JB . 2010 Ecological opportunity and the rate of morphological evolution in the diversification of Greater Antillean anoles. Evolution 64: 2731–2745. 20455931 10.1111/j.1558-5646.2010.01026.x

[nph16176-bib-0046] Manceau M , Lambert A , Morlon H . 2017 A unifying comparative phylogenetic framework including traits coevolving across interacting lineages. Systematic Biology 66: 551–568. 28003533 10.1093/sysbio/syw115

[nph16176-bib-0047] Maron JL , Agrawal AA , Schemske DW . 2019 Plant–herbivore coevolution and plant speciation. Ecology 100: e02704. 30916391 10.1002/ecy.2704

[nph16176-bib-0048] Marquis RJ , Salazar D , Baer C , Reinhardt J , Priest G , Barnett K . 2016 Ode to Ehrlich and Raven or how herbivorous insects might drive plant speciation. Ecology 97: 2939–2951. 27870033 10.1002/ecy.1534

[nph16176-bib-0049] Marussich WA , Machado CA . 2007 Host‐specificity and coevolution among pollinating and nonpollinating New World fig wasps. Molecular Ecology 16: 1925–1946. 17444902 10.1111/j.1365-294X.2007.03278.x

[nph16176-bib-0050] Navaud O , Barbacci A , Taylor A , Clarkson JP , Raffaele S . 2018 Shifts in diversification rates and host jump frequencies shaped the diversity of host range among Sclerotiniaceae fungal plant pathogens. Molecular Ecology 27: 1309–1323. 29421852 10.1111/mec.14523 PMC5900718

[nph16176-bib-0051] van der Niet T , Peakall R , Johnson SD . 2014 Pollinator‐driven ecological speciation in plants: new evidence and future perspectives. Annals of Botany 113: 199–211. 24418954 10.1093/aob/mct290 PMC3890394

[nph16176-bib-0052] Nuismer SL , Harmon LJ . 2015 Predicting rates of interspecific interaction from phylogenetic trees. Ecology Letters 18: 17–27. 25349102 10.1111/ele.12384

[nph16176-bib-0053] Nyman T . 2010 To speciate, or not to speciate? Resource heterogeneity, the subjectivity of similarity, and the macroevolutionary consequences of niche‐width shifts in plant‐feeding insects. Biological Reviews 85: 393–411. 20002390 10.1111/j.1469-185X.2009.00109.x

[nph16176-bib-0054] Paradis E , Claude J , Strimmer K . 2004 ape: analyses of phylogenetics and evolution in R language. Bioinformatics 20: 289–290. 14734327 10.1093/bioinformatics/btg412

[nph16176-bib-0055] Patel A . 1996 Variation in a mutualism: phenology and the maintenance of gynodioecy in two Indian fig species. Journal of Ecology 84: 667–680.

[nph16176-bib-0056] Pellmyr O , Leebens‐Mack J , Huth CJ . 1996 Non‐mutualistic yucca moths and their evolutionary consequences. Nature 380: 155–156. 8600388 10.1038/380155a0

[nph16176-bib-0057] Peng YQ , Duan ZB , Yang DR , Rasplus J‐Y . 2008 Co‐occurrence of two *Eupristina* species on *Ficus altissima* in Xishuangbanna, SW China. Symbiosis 45: 9–14.

[nph16176-bib-0058] Pereira RAS , do Parado AP . 2005 Non‐pollinating wasps distort the sex ratio of pollinating fig wasps. Oikos 110: 613–619.

[nph16176-bib-0059] Purcell MF , Thornhill AH , Wallenius TC , Yeates DK , Rowell DM . 2018 Plant host relationships of three lineages of the gall‐inducing fly *Fergusonina* Malloch (Diptera: Fergusoninidae) on *Eucalyptus* L'Herit. Arthropod–Plant Interactions 12: 133–145.

[nph16176-bib-0060] R Core Team . 2018 R: a language and environment for statistical computing. Vienna, Austria: R Foundation for Statistical Computing.

[nph16176-bib-0061] Raja S , Suleman N , Quinnell RJ , Compton SG . 2015 Interactions between pollinator and non‐pollinator fig wasps: correlations between their numbers can be misleading. Entomological Science 18: 230–236.

[nph16176-bib-0062] Rasplus J‐Y , Kerdelhué C , Le Clainche I , Mondor G . 1998 Molecular phylogeny of fig wasps Agaonidae are not monophyletic. Comptes Rendus de l'Académie des Sciences – Series III – Sciences de la Vie 321: 517–527. 10.1016/s0764-4469(98)80784-1 9841095

[nph16176-bib-0063] Rønsted N . 2005 60 million years of co‐divergence in the fig–wasp symbiosis. Proceedings of the Royal Society of London. Series B: Biological Sciences 272: 2593–2599. 16321781 10.1098/rspb.2005.3249 PMC1559977

[nph16176-bib-0064] Segar ST , Pereira RAS , Compton SG , Cook JM . 2013 Convergent structure of multitrophic communities over three continents. Ecology Letters 16: 1436–1445. 24134201 10.1111/ele.12183

[nph16176-bib-0065] Silvieus SI , Clement WL , Weiblen GD . 2008 Cophylogeny of figs, pollinators, gallers and parasitoids In: Tilmon KJ, ed. Specialization, speciation and radiation: the evolutionary biology of herbivorous insects. Berkeley, CA, USA: University of California Press, 225–239.

[nph16176-bib-0066] Sweet AD , Boyd BM , Johnson KP . 2016 Cophylogenetic patterns are uncorrelated between two lineages of parasites on the same hosts. Biological Journal of Linnean Society 118: 813–828.

[nph16176-bib-0067] Verkerke W . 1989 Structure and function of the fig. Experientia 45: 612–622.

[nph16176-bib-0068] Weiblen GD . 2002 How to be a fig wasp. Annual Review of Entomology 47: 299–330. 10.1146/annurev.ento.47.091201.145213 11729077

[nph16176-bib-0069] Weiblen GD , Bush GL . 2002 Speciation in fig pollinators and parasites. Molecular Ecology 11: 1573–1578. 12144676 10.1046/j.1365-294x.2002.01529.x

[nph16176-bib-0070] Weiblen GD , Yu DW , West SA . 2001 Pollination and parasitism in functionally dioecious figs. Proceedings of the Royal Society of London. Series B: Biological Sciences 268: 651–659. 11297184 10.1098/rspb.2000.1389 PMC1088653

[nph16176-bib-0071] West SA , Herre EA , Windsor DM , Green PRS . 1996 The ecology and evolution of the New World non‐pollinating fig wasp communities. Journal of Biogeography 23: 447–458.

[nph16176-bib-0072] Wu T , Dunn DW , Hu HY , Niu LM , Xiao JH , Pan XL , Feng G , Fu YG , Huang DW . 2013 The occurrence of fig wasps in the fruits of female gynodioecious fig trees. Acta Oecologica 46: 33–38.

[nph16176-bib-0073] Xu L , Yang DR , Peng YQ , Wei ZD . 2005 *Ficus* and the wasp community within syconia in Xishuangbanna. Forest Research 18: 497–503.

[nph16176-bib-0074] Yang LY , Machado CA , Dang XD , Peng YQ , Yang DR , Zhang DY , Liao WJ . 2015 The incidence and pattern of copollinator diversification in dioecious and monoecious figs. Evolution 69: 294–304. 25495152 10.1111/evo.12584 PMC4328460

[nph16176-bib-0075] Yang DR , Xu L , Peng YQ , Wei ZD , Duan ZB . 2004 Species composition and diversity of fig wasps and figs in Yunnan. Biodiversity Science 12: 611–617.

[nph16176-bib-0076] Yoder JB , Nuismer SL . 2010 When does coevolution promote diversification? American Naturalist 176: 802–817. 10.1086/657048 20950142

[nph16176-bib-0077] Zhang FP , Peng YQ , Yang DR . 2008 Coevolution between two internal ovipositing fig wasps and host *Ficus curtipes* . Journal of Plant Ecology (Chinese Version) 32: 768–775 (in Chinese with English abstract).

