# Supplementary material for: The nature of interspecific interactions and co‐diversification patterns, as illustrated by the fig microcosm
Source: New Phytol. 2019 Oct 4;224(3):1304–15. doi: 10.1111/nph.16176 (PMC6856861; doi:10.1111/nph.16176)
Supplement: Supplementary file 1 — Fig. S1 Co‐phylogenetic associations of Ficus and galling wasps, and pollinating and galling wasp species, excluding secondary galling species. Fig. S2 The frequencies of different types of phylogenetic events inferred by Jane for pairwise comparisons of the phylogenies of Ficus, pollinating wasps and galling wasps, excluding secondary galling species. Fig. S3 Phylogenetic trees for Ficus, pollinating wasps and galling wasps. Notes S1 Summary of phylogenetic results for Ficus, pollinating and galling wasps. Table S1 Ficus and fig wasp species included in the phylogenetic and co‐phylogenetic analyses. Table S2 Taxonomic affinity, sexual systems and GenBank accession numbers of Ficus species involved in this study. Table S3 Ecological associations and roles, collection information and GenBank accession numbers for wasps included in this study. [file NPH-224-1304-s001.pdf]

***New Phytologist* Supporting Information**

Article title: The nature of interspecific interactions and codiversification patterns, as illustrated by the fig microcosm
Authors: Ai-Ying Wang, Yan-Qiong Peng, Lawrence D. Harder, Jian-Feng Huang, Da-Rong Yang, Da-Yong Zhang, Wan-Jin Liao
Article acceptance date: 02 September 2019

The following Supporting Information is available for this article:

Notes S1 Summary of phylogenetic results for *Ficus*, pollinating and galling wasps.

Fig. S1 Cophylogenetic associations between *Ficus* and galling wasps, and pollinating and galling wasps, excluding secondary galling species.

Fig. S2 The frequencies of different types of cophylogenetic events inferred by Jane for pairwise comparisons of the phylogenies of *Ficus*, pollinating wasps and galling wasps, excluding secondary galling species.

Fig. S3 Phylogenetic trees for *Ficus*, pollinating wasps and galling wasps.

Table S1 *Ficus* and fig wasp species included in the phylogenetic and cophylogenetic analyses. Alphabetic footnotes identify subgenus for *Ficus* species, and family/subfamily for wasp species.

Table S2 Taxonomic affinity, sexual systems and GenBank accession numbers of *Ficus* species involved in this study.

Table S3 Ecological associations and roles, collection information and GenBank accession numbers for wasps included in this study.

Notes S1 Summary of phylogenetic results of *Ficus*, pollinating and galling wasps.

***Ficus* –** Phylogenetic inference for the 26 fig species was based on 27, 43, 34 and 28 haplotypes for ETS, *G3pdh*, ITS and *trnL-F*, respectively, with a total sequence length of 2875 bp. All species were represented by sequences for all four genes, except ITS for *Ficus racemosa* (Table S2). The sampled *Ficus* species constitute a monophyletic clade (Fig. 2a, Supporting Information Fig. S3a), as inferred by Bayesian phylogenetic analysis (substitution models HKY+G for ETS, GTR+G for the other three genes: posterior probability = 1). Within this clade, subgenus *Urostigma* was recovered as polyphyletic, which is consistent with Cruaud *et al.*'s (2012) more extensive analysis. Subgenus *Sycomorus* was inferred as monophyletic with a posterior probability of 0.99, except for *F. racemosa*. Dioecious and monoecious figs do not represent separate monophyletic clades.

Pollinating wasps – For the 26 species of pollinating wasps and two cheating species, sequences were obtained for 129 individuals for 18S rRNA, 96 individuals for 28S rRNA and 111 individuals for *COI* (Table S2). The total aligned sequence length was 2107 bp. Two species were not represented by 28S rRNA sequences and three species were not represented by *COI* (Table S3). The phylogenetic relationships inferred for the 28 pollinating wasp species (GTR+G+I substitution model) were mostly consistent with those identified by Cruaud *et al.*'s (2012) global analysis (Fig. 2a, Supporting Information Fig. S3b). *Ceratosolen* and *Kradibia* formed monophyletic groups. In contrast, our analysis infers *Eupristina* as polyphyletic, as the two species from *Ficus microcarpa* ally most closely with *Deliagaon* and *Maniella*. This result differs from that of Cruaud *et al.* (2012), who inferred six *Eupristina* species as monophyletic, including four in our sample; however, they did not sample wasps from *F. microcarpa*. The cheating wasp species, *Eupristina* sp. 1 from *F. altissima* and *Eupristina* sp. 3 from

F. microcarpa, were each the closest relative of the pollinating wasp species using the same *Ficus* species (Supporting Information Fig. S3b, blue symbols).

Galling wasps – For the 33 galling wasp species, gene sequences included 106 individuals for 18S rRNA, 90 individuals for 28S rRNA and 78 individuals for *COI*, with a total aligned sequence length of 2083 bp (Table S2). Three and two species were not represented by sequences of 28S rRNA and *COI*, respectively (Table S3). For the galling wasps, Bayesian analysis (GTR+G substitution model) inferred subfamily Epichrysomallinae (Fig. 2b, c, orange symbols), Otitesellinae (Fig. 2b, c, green symbols) and genus *Sycophaga* (Fig. 2b, c, purple symbols) as monophyletic groups with > 0.99 posterior probabilities. However, most genera within subfamilies were not inferred as monophyletic groups. Four *Acophila* species and five *Walkerella* species formed a monophyletic group, respectively, with > 0.97 posterior probability (Fig. 2b, Supporting Information Fig. S3c).

References

Cruaud A, Ronsted N, Chantarasuwan B, Chou LS, Clement WL, Couloux A, Cousins B, Genson G, Harrison RD, Hanson PE, *et al.* 2012. An extreme case of plant-insect codiversification: figs and fig-pollinating wasps. *Systematic Biology* **61**: 1029-1047.

Fig. S1 Cophylogenetic patterns between (a) *Ficus* and galling wasps and (b) pollinating and galling wasps, excluding secondary galling species. Red and black tip labels for *Ficus* distinguish dioecious and monoecious species, respectively. Subfamily Epichrysomallinae, Otitesellinae and Sycophaginae were shown in orange, green and purple in (a) and (b). Solid lines connecting species in the left- and right-hand phylogenetic trees indicate observed associations. Line thickness varies positively with the contribution of a particular association to overall phylogenetic congruence. The "ex …" labels identify the host fig species of individual wasp species. Abbreviations in (a) and (b) for figs: *F*. = *Ficus*. Abbreviations in (b) for fig pollinating wasps: *B*. = *Blastophaga*, *C*. = *Ceratosolen*, *D*. = *Deliagaon*, *E*. = *Eupristina*, *H*. = *Hederagaon*, *K*. = *Kradibia*, *P*. = *Platyscapa*, *U*. = *Umagaon*, *W*. = *Waterstoniella*. Abbreviations in (a) and (b) for galling wasps: *Ac*. = *Acophila*, *Cam*. = *Camarothorax*, *Mi*. = *Micranisa*, *Neo*. = *Neosycophila*, *Od*. = *Odontofroggatia*, *Sb*. = *Sycobia*, *Syg*. = *Sycophaga*, *Sym*. = *Sycophilomorpha*, *Wa*. = *Walkerella*.

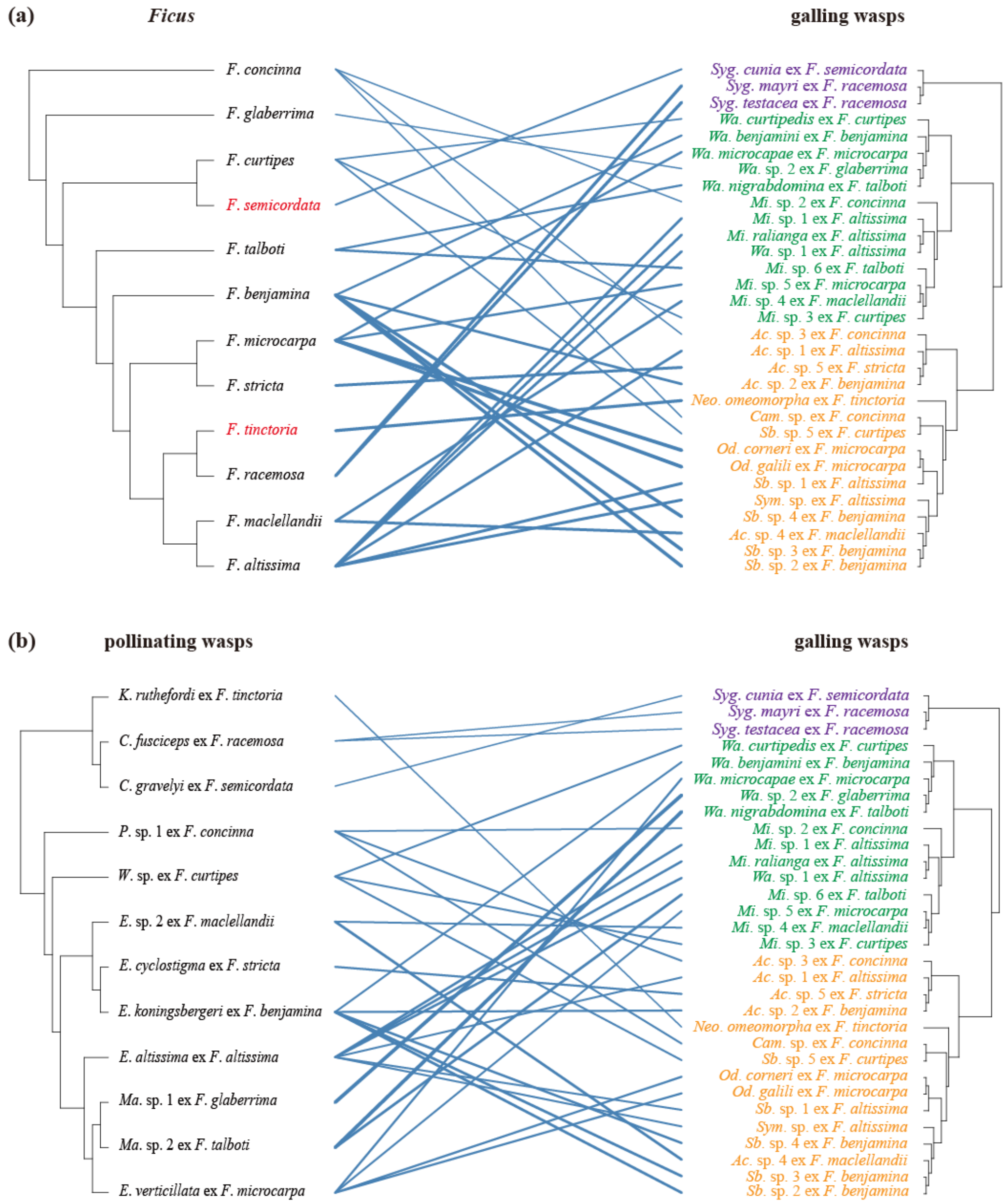
(a)
Ficus
galling wasps
F. concinna
F. glaberrima
F. curtipes
F. semicordata
F. talboti
F. benjamina
F. microcarpa
F. stricta
F. tinctoria
F. racemosa
F. maclellandii
F. altissima
Syg. cunia ex F. semicordata
Syg. mayri ex F. racemosa
Syg. testacea ex F. racemosa
Wa. curtipedis ex F. curtipes
Wa. benjamini ex F. benjamina
Wa. microcapae ex F. microcarpa
Wa. sp. 2 ex F. glaberrima
Wa. nigrabdomina ex F. talboti
Mi. sp. 2 ex F. concinna
Mi. sp. 1 ex F. altissima
Mi. raliang ex F. altissima
Wa. sp. 1 ex F. altissima
Mi. sp. 6 ex F. talboti
Mi. sp. 5 ex F. microcarpa
Mi. sp. 4 ex F. maclellandii
Mi. sp. 3 ex F. curtipes
Ac. sp. 3 ex F. concinna
Ac. sp. 1 ex F. altissima
Ac. sp. 5 ex F. stricta
Ac. sp. 2 ex F. benjamina
Neo. omeomorpha ex F. tinctoria
Cam. sp. ex F. concinna
Sb. sp. 5 ex F. curtipes
Od. corneri ex F. microcarpa
Od. galili ex F. microcarpa
Sb. sp. 1 ex F. altissima
Sym. sp. ex F. altissima
Sb. sp. 4 ex F. benjamina
Ac. sp. 4 ex F. maclellandii
Sb. sp. 3 ex F. benjamina
Sb. sp. 2 ex F. benjamina
(b)
pollinating wasps
galling wasps
K. ruthefordi ex F. tinctoria
C. fusciceps ex F. racemosa
C. gravelyi ex F. semicordata
P. sp. 1 ex F. concinna
W. sp. ex F. curtipes
E. sp. 2 ex F. maclellandii
E. cyclostigma ex F. stricta
E. koningsbergeri ex F. benjamina
E. altissima ex F. altissima
Ma. sp. 1 ex F. glaberrima
Ma. sp. 2 ex F. talboti
E. verticillata ex F. microcarpa
Syg. cunia ex F. semicordata
Syg. mayri ex F. racemosa
Syg. testacea ex F. racemosa
Wa. curtipedis ex F. curtipes
Wa. benjamini ex F. benjamina
Wa. microcapae ex F. microcarpa
Wa. sp. 2 ex F. glaberrima
Wa. nigrabdomina ex F. talboti
Mi. sp. 2 ex F. concinna
Mi. sp. 1 ex F. altissima
Mi. raliang ex F. altissima
Wa. sp. 1 ex F. altissima
Mi. sp. 6 ex F. talboti
Mi. sp. 5 ex F. microcarpa
Mi. sp. 4 ex F. maclellandii
Mi. sp. 3 ex F. curtipes
Ac. sp. 3 ex F. concinna
Ac. sp. 1 ex F. altissima
Ac. sp. 5 ex F. stricta
Ac. sp. 2 ex F. benjamina
Neo. omeomorpha ex F. tinctoria
Cam. sp. ex F. concinna
Sb. sp. 5 ex F. curtipes
Od. corneri ex F. microcarpa
Od. galili ex F. microcarpa
Sb. sp. 1 ex F. altissima
Sym. sp. ex F. altissima
Sb. sp. 4 ex F. benjamina
Ac. sp. 4 ex F. maclellandii
Sb. sp. 3 ex F. benjamina
Sb. sp. 2 ex F. benjamina

Fig. S2 The frequencies of different types of cophylogenetic events (cospeciation, duplication, association switch, association loss) inferred by Jane for pairwise comparisons of the phylogenies of (a) pollinating wasps and galling wasps in the context of *Ficus* evolution, (b) *Ficus* and galling wasps in the context of pollinator evolution, and (c) galling wasps in the context of *Ficus* and pollinating wasp evolution, excluding secondary galling species respectively. X|Y denotes that clade Y was the reference clade and clade X was comparator the clade, and error bars indicate standard deviations for cases in which Jane identified several different sets of outcomes associated with the same minimal cost.

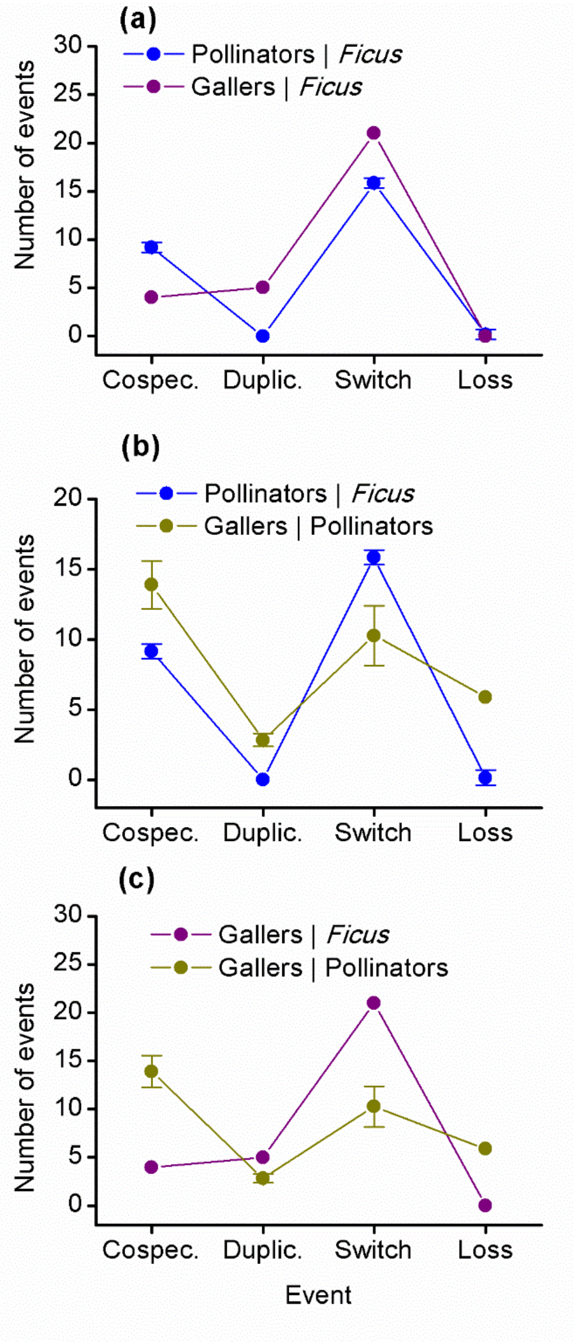

Fig. S3 Phylogenetic trees for (a) *Ficus*, (b) pollinating wasps, (c) galling wasps and (d) galling wasps without secondary gallers. Bayesian posterior probabilities are shown above branches. Scale bars below each panel represent the number of substitution per site. Outgroups are shown in grey. In panel (a), red and black tip labels distinguish dioecious and monoecious fig species, respectively. Two cheater wasp species were shown in blue in (b). In panels b – d coloured boxes distinguish Kradibiinae (red in panel b), Epichrysomallinae (green in panels c and d), Otitesellinae (red in panels c and d) and *Sycophaga* (yellow in panels c and d). Abbreviations: *F*. = *Ficus*; *B*. = *Blastophaga*, *C*. = *Ceratosolen*, *D*. = *Deliagaon*, *E*. = *Eupristina*, *H*. = *Hederagaon*, *K*. = *Kradibia*, *Ma.* = *Maniella*, *P*. = *Platyscapa*, *U*. = *Umagaon*, *W*. = *Waterstoniella*; *Ac*. = *Acophila*, *Cam*. = *Camarothorax*, *Dia*. = *Diaziella*, *Lip*. = *Lipothymus*, *Mi*. = *Micranisa*, *Neo*. = *Neosycophila*, *Od*. = *Odontofroggatia*, *Sb*. = *Sycobia*, *Syg*. = *Sycophaga*, *Sym*. = *Sycophilomorpha*, *Wa*. = *Walkerella*. "ex …" identifies the host fig species for each wasp species.

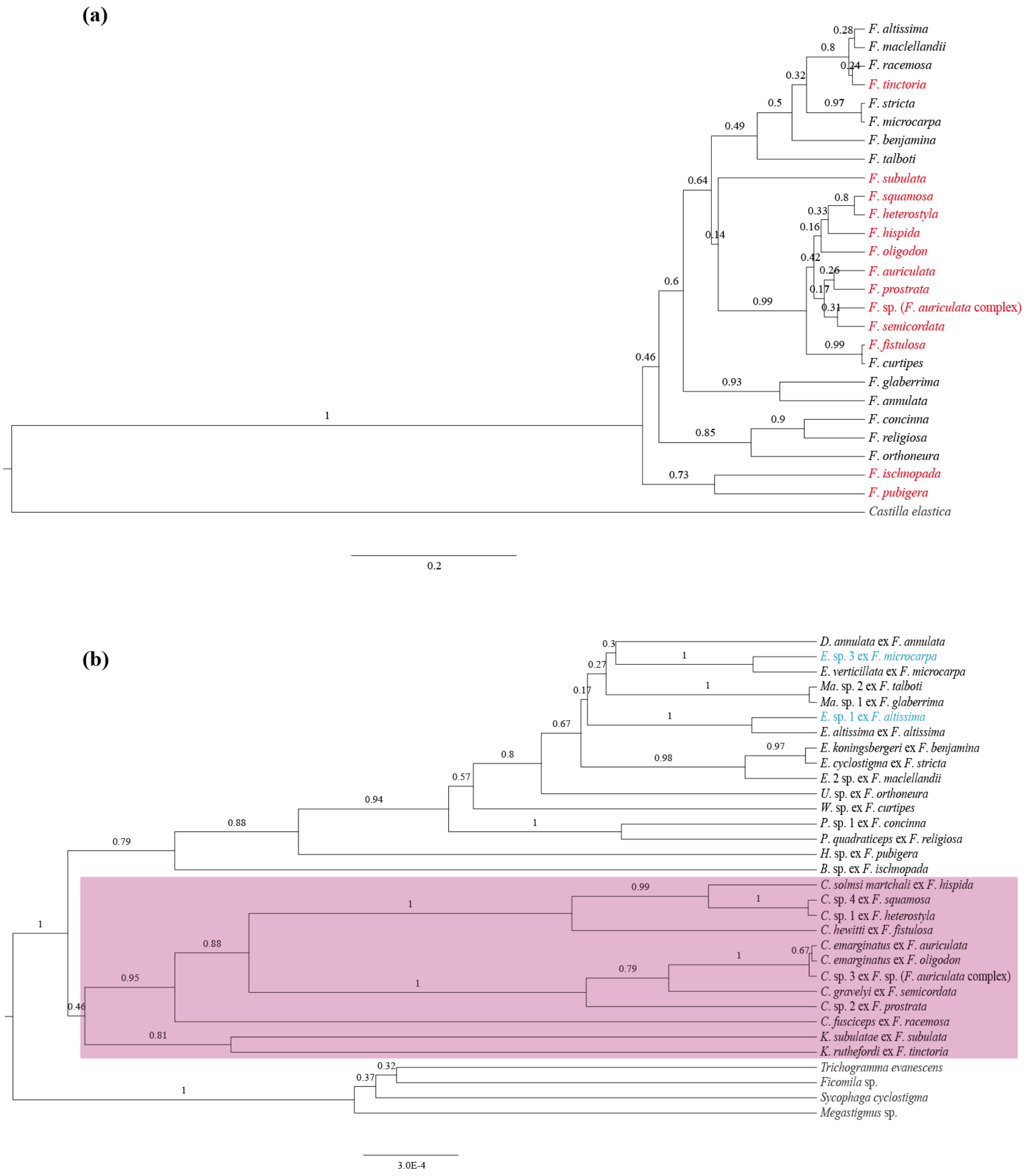
(a)
F. altissima
F. maclellandii
F. racemosa
F. tinctoria
F. stricta
F. microcarpa
F. benjamina
F. talboti
F. subulata
F. squamosa
F. heterostyla
F. hispida
F. oligodon
F. auriculata
F. prostrata
F. sp. (F. auriculata complex)
F. semicordata
F. fistulosa
F. curtipes
F. glaberrima
F. annulata
F. concinna
F. religiosa
F. orthoneura
F. ischnopada
F. pubigera
Castilla elastica
0.2
(b)
D. annulata ex F. annulata
E. sp. 3 ex F. microcarpa
E. verticillata ex F. microcarpa
Ma. sp. 2 ex F. talboti
Ma. sp. 1 ex F. glaberrima
E. sp. 1 ex F. altissima
E. altissima ex F. altissima
E. koningsbergeri ex F. benjamina
E. cyclostigma ex F. stricta
E. 2 sp. ex F. maclellandii
U. sp. ex F. orthoneura
W. sp. ex F. curtipes
P. sp. 1 ex F. concinna
P. quadraticeps ex F. religiosa
H. sp. ex F. pubigera
B. sp. ex F. ischnopada
C. solmsi martchali ex F. hispida
C. sp. 4 ex F. squamosa
C. sp. 1 ex F. heterostyla
C. hewitti ex F. fistulosa
C. emarginatus ex F. auriculata
C. emarginatus ex F. oligodon
C. sp. 3 ex F. sp. (F. auriculata complex)
C. gravelyi ex F. semicordata
C. sp. 2 ex F. prostrata
C. fusciceps ex F. racemosa
K. subulatae ex F. subulata
K. ruthefordi ex F. tinctoria
Trichogramma evanescens
Ficomila sp.
Sycophaga cyclostigma
Megastigmus sp.
3.0E-4

(c)

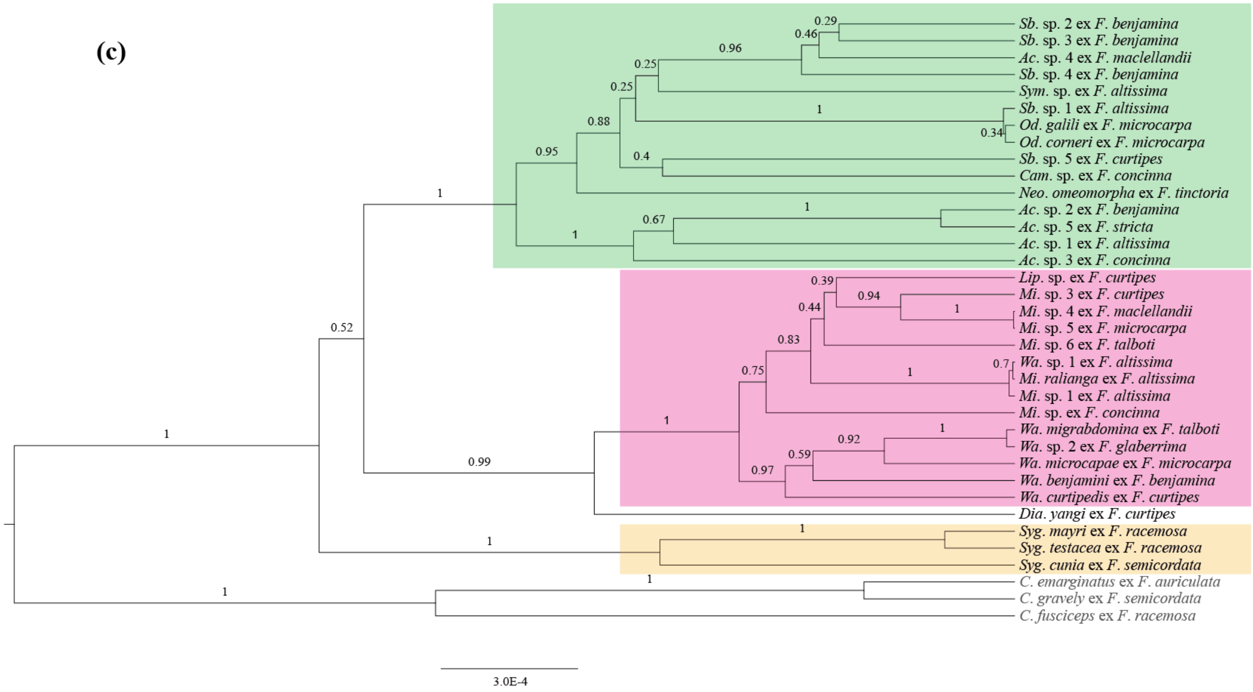

Sb. sp. 2 ex F. benjamina
Sb. sp. 3 ex F. benjamina
Ac. sp. 4 ex F. maclellandii
Sb. sp. 4 ex F. benjamina
Sym. sp. ex F. altissima
Sb. sp. 1 ex F. altissima
Od. galili ex F. microcarpa
Od. corneri ex F. microcarpa
Sb. sp. 5 ex F. curtipes
Cam. sp. ex F. concinna
Neo. omeomorpha ex F. tinctoria
Ac. sp. 2 ex F. benjamina
Ac. sp. 5 ex F. stricta
Ac. sp. 1 ex F. altissima
Ac. sp. 3 ex F. concinna
Lip. sp. ex F. curtipes
Mi. sp. 3 ex F. curtipes
Mi. sp. 4 ex F. maclellandii
Mi. sp. 5 ex F. microcarpa
Mi. sp. 6 ex F. talboti
Wa. sp. 1 ex F. altissima
Mi. ralianga ex F. altissima
Mi. sp. 1 ex F. altissima
Mi. sp. ex F. concinna
Wa. migrabdomina ex F. talboti
Wa. sp. 2 ex F. glaberrima
Wa. microcapae ex F. microcarpa
Wa. benjamini ex F. benjamina
Wa. curtipedis ex F. curtipes
Dia. yangi ex F. curtipes
Syg. mayri ex F. racemosa
Syg. testacea ex F. racemosa
Syg. cunia ex F. semicordata
C. emarginatus ex F. auriculata
C. gravely ex F. semicordata
C. fusciceps ex F. racemosa
3.0E-4

(d)

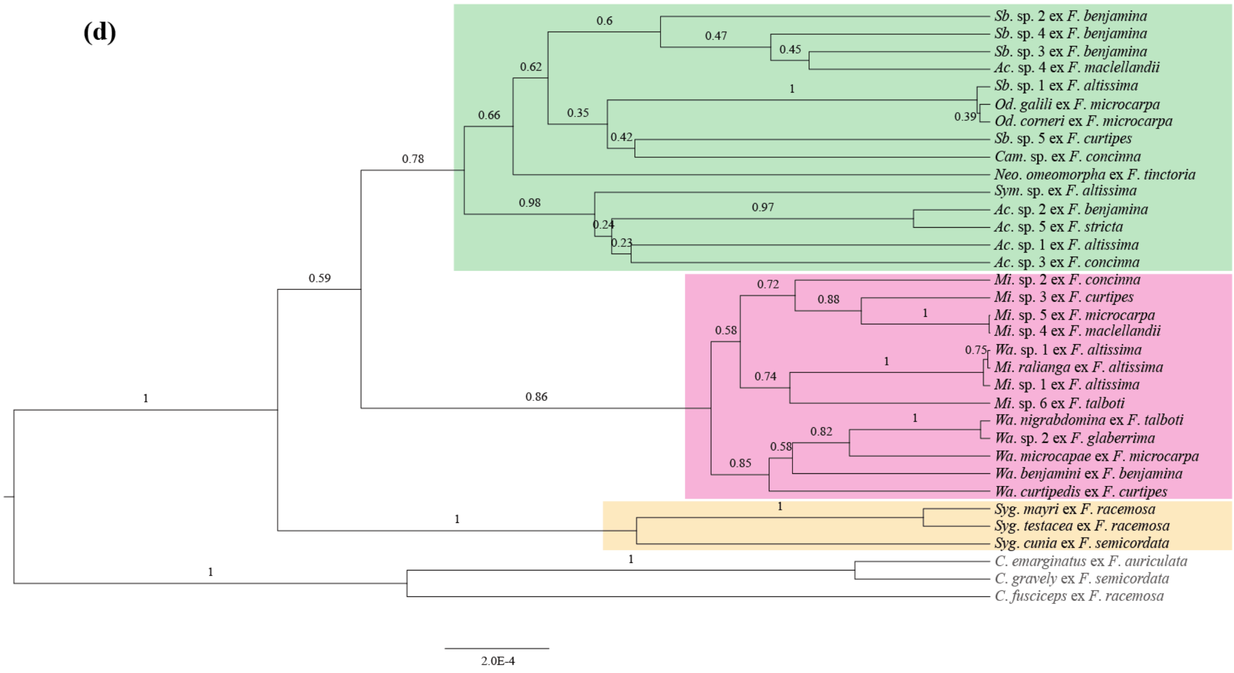

Sb. sp. 2 ex F. benjamina
Sb. sp. 4 ex F. benjamina
Sb. sp. 3 ex F. benjamina
Ac. sp. 4 ex F. maclellandii
Sb. sp. 1 ex F. altissima
Od. galili ex F. microcarpa
Od. corneri ex F. microcarpa
Sb. sp. 5 ex F. curtipes
Cam. sp. ex F. concinna
Neo. omeomorpha ex F. tinctoria
Sym. sp. ex F. altissima
Ac. sp. 2 ex F. benjamina
Ac. sp. 5 ex F. stricta
Ac. sp. 1 ex F. altissima
Ac. sp. 3 ex F. concinna
Mi. sp. 2 ex F. concinna
Mi. sp. 3 ex F. curtipes
Mi. sp. 5 ex F. microcarpa
Mi. sp. 4 ex F. maclellandii
Wa. sp. 1 ex F. altissima
Mi. ralianga ex F. altissima
Mi. sp. 1 ex F. altissima
Mi. sp. 6 ex F. talboti
Wa. nigrabdomina ex F. talboti
Wa. sp. 2 ex F. glaberrima
Wa. microcapae ex F. microcarpa
Wa. benjamini ex F. benjamina
Wa. curtipedis ex F. curtipes
Syg. mayri ex F. racemosa
Syg. testacea ex F. racemosa
Syg. cunia ex F. semicordata
C. emarginatus ex F. auriculata
C. gravely ex F. semicordata
C. fusciceps ex F. racemosa
2.0E-4

Table S1 *Ficus* and fig wasp species included in the phylogenetic and cophylogenetic analyses. Alphabetic footnotes identify subgenus for *Ficus* species, and family/subfamily for wasp species.

Ficus	**Abundance**	**Pollinating wasps**	**Galling wasps**
**Dioecious**			
F. auriculata[Sco]	common	*Ceratosolen emarginatus*[Ak]	-[1]
F. fistulosa[Sco]	sporadic	*Ceratosolen hewitti*[Ak]	-
F. heterostyla[Sco]	common	*Ceratosolen* sp. 1[Ak]	-
F. hispida[Sco]	common	*Ceratosolen solmsi martchali*[Ak]	-
F. ischnopada[Fic]	sporadic	*Blastophaga* sp.[Aa]	-
F. oligodon[Sco]	common	*Ceratosolen emarginatus*[Ak]	-
F. sp. (*F. auriculata* complex) [Sco]	sporadic	*Ceratosolen* sp. 3[Ak]	-
F. prostrata[Sco]	sporadic	*Ceratosolen* sp. 2[Ak]	-
F. pubigera[Sno]	sporadic	*Hederagaon* sp.[Aa]	-
F. semicordata[Sco]	common	*Ceratosolen gravelyi*[Ak]	*Sycophaga cunia*[As]
F. squamosa[Sco]	common	*Cerasotolen* sp. 4[Ak]	-
F. subulata[Sci]	common	*Kradibia subulatae*[Ak]	-
F. tinctoria[Sci]	common	*Kradibia ruthefordi*[Ak]	*Neosycophila omeomorpha*[Pe]
**Monoecious**			
F. altissima[Uro]	common	*Eupristina altissima* [Aa]<br>*Eupristina* sp. 1[2, Aa]	*Acophila* sp. 1[Pe]<br>*Micranisa ralianga*[Po]<br>*Micranisa* sp. 1[Po]<br>*Sycobia* sp. 1[Pe]<br>*Sycophilomorpha* sp.[Pe]<br>*Walkerella* sp. 1[Po]
F. annulata[Uro]	sporadic	*Deliagaon annulatae*[Aa]	-
F. benjamina[Uro]	common	*Eupristina koningsbergeri* [Aa]	*Acophila* sp. 2[Pe]<br>*Sycobia* sp. 2[Pe]<br>*Sycobia* sp. 3[Pe]<br>*Sycobia* sp. 4[Pe]<br>*Walkerella benjamini*[Po]
F. concinna[Uro]	common	*Platyscapa* sp. 1[Aa]	*Acophila* sp. 3[Pe]<br>*Camarothorax* sp.[Pe]<br>*Micranisa* sp. 2[Po]
F. curtipes[Uro]	common	*Waterstoniella* sp.[Aa]	*Diaziella yangi*[Ps]<br>*Lipothymus* sp.[Po]<br>*Micranisa* sp. 3[Po]<br>*Sycobia* sp. 5[Pe]<br>*Walkerella curtipedis*[Po]
F. glaberrima[Uro]	sporadic	*Maniella* sp. 1[Aa]	*Walkerella* sp. 2[Po]
F. maclellandii[Uro]	common	*Eupristina* sp. 2[Aa]	*Acophila* sp. 4[Pe]<br>*Micranisa* sp. 4[Po]
F. microcarpa[Uro]	common	*Eupristina verticillata*[Aa]<br>*Eupristina* sp. 3[2, Aa]	*Micranisa* sp. 5[Po]<br>*Odontofroggatia corneri*[Pe]<br>*Odontofroggatia galili*[Pe]

			Walkerella microcapae[Po]
F. orthoneura[Uro]	sporadic	*Umagaon* sp.[Aa]	-
F. racemosa[Sco]	common	*Ceratosolen fusciceps*[Ak]	*Sycophaga mayri*[As]<br>*Sycophaga testacea*[As]
F. religiosa[Uro]	common	*Platyscapa quadraticeps*[Aa]	-
F. stricta[Uro]	sporadic	*Eupristina cyclostigma*[Aa]	*Acophila* sp. 5[Pe]
F. talboti[Uro]	sporadic	*Maniella* sp. 2[Aa]	*Micranisa* sp. 6[Po]<br>*Walkerella nigrabdomina*[Po]

[Fic] – Ficus, [Sci] – Sycidium, [Sco] – Sycomorus, [Sno] – Synoecia, [Uro] – Urostigma

[Aa] – Agaonidae/Agaoninae, [Ak] – Agaonidae/Kradibiinae, [As] – Agaonidae/Sycophaginae, [Pe] – Pteromalidae/Epichrysomallinae, [Po] – Pteromalidae/Otitesellinae, [Ps] – Pteromalidae/Sycoecinae

[1] A dash, “-”, indicates that no galling wasps occupied synconia sampled from the associated fig species.

[2] This species acted as a cheater.

Table S2 Taxonomic affinity, sexual systems and GenBank accession numbers of fig species involved in this study. Sequences downloaded from NCBI are indicated in bold font.

Subgenus and species	Sexual system	ITS	ETS	*G3pdh*	*trnL-F*
**Subg. *Sycomorus***					
Ficus auriculata Lour.	Dioecy	MK472748<br>MK472749<br>MK472750	MK472721	MK507022	MK472783
Ficus fistulosa Reinw. ex Blume	Dioecy	MK472755	MK472726	MK507035	MK472790
Ficus heterostyla Merr.	Dioecy	MK472757	MK472728	MK507037	MK472792
Ficus hispida L. f.	Dioecy	MK472758<br>MK472759<br>MK472760<br>MK472761	MK472729	MK507038	MK472793
Ficus oligodon Miq.	Dioecy	MK472765	MK472733	MK507046	MK472797
Ficus sp. (*F. auriculata* complex)	Dioecy	MK472780	MK472745	MK507058<br>MK507059<br>MK507060<br>MK507061<br>MK507062	MK472809
Ficus prostrata (Wall ex Miq.) Miq	Dioecy	MK472768	MK472736	MK507049	MK472800
Ficus racemosa L.	Monoecy	-	MK472738	MK507051	MK472802
Ficus semicordata Buch-Ham ex Sm	Dioecy	MK472771	MK472740	MK507053	MK472804
Ficus squamosa Roxb.	Dioecy	MK472772	MK472741	MK507054	MK472805
**Subg. *Urostigma***					
Ficus altissima Blume	Monoecy	MK472746	MK472718<br>MK472719	MK507015<br>MK507016	MK472781
Ficus annulata Blume	Monoecy	MK472747	MK472720	MK507017<br>MK507018<br>MK507019<br>MK507020<br>MK507021	MK472782
Ficus benjamina L.	Monoecy	MK472751	MK472722	MK507023	MK472784<br>MK472785<br>MK472786
Ficus concinna (Miq.) Miq.	Monoecy	MK472752	MK472723	MK507024<br>MK507025<br>MK507026<br>MK507027<br>MK507028	MK472787
Ficus curtipes Corner	Monoecy	MK472754	MK472725	MK507034	MK472789
Ficus glaberrima Blume	Monoecy	MK472756	MK472727	MK507036	MK472791
Ficus maclellandii King	Monoecy	MK472763	MK472731	MK507040	MK472795
Ficus microcarpa L.	Monoecy	MK472764	MK472732	MK507041<br>MK507042<br>MK507043<br>MK507044<br>MK507045	MK472796

Subgenus and species	Sexual system	ITS	ETS	*G3pdh*	*trnL-F*
Ficus orthoneura Lévleillé & Vaniot	Monoecy	MK472766	MK472734	MK507047	MK472798
Ficus religiosa L.	Monoecy	MK472770	MK472739	MK507052	MK472803
Ficus stricta (Miq.) Miq.	Monoecy	MK472773	MK472742	MK507055	MK472806
Ficus talboti (Blume)	Monoecy	MK472767	MK472735	MK507048	MK472799
**Subg. *Sycidium***					
Ficus subulata Blume	Dioecy	MK472774<br>MK472775<br>MK472776<br>MK472777<br>MK472778	MK472743	MK507056	MK472807
Ficus tinctoria Forst. f.	Dioecy	MK472779	MK472744	MK507057	MK472808
**Subg. *Ficus***					
Ficus ischnopada Miq.	Dioecy	MK472762	MK472730	MK507039	MK472794
**Subg. *Synoecia***					
Ficus pubigera (Miq.) Miq.	Dioecy	MK472769	MK472737	MK507050	MK472801
**OUTGROUP**					
Castilla elastica Sessé		**AY730143**	**AY730232**	**EF092327**	**AF501603**

Table S3 Ecological associations and roles, collection information and GenBank accession numbers for wasps included in this study. Sequences downloaded from NCBI are indicated in bold font.

Family, subfamily and species	Host *Ficus* species	Ecological role	Collection period	Number of individuals	18S	28S	*COI*
**Agaonidae**							
**Agaoninae**							
Blastophaga sp.	*F. ischnopada*	pollinator	2008-2018	1	**JN102917**	**JN102696**	**JN103262**
					MK474058		
Deliagaon annulatae Wiebes, 1977	*F. annulata*	pollinator	2008-2018	8	MK474013	MK495117	MK543400
					MK474014	MK495118	MK543401
					MK474015	MK495119	MK543402
					MK474016		MK543403
					MK474017		MK543404
					MK474018		MK543405
					MK474019		MK543406
					MK474020		MK543407
Eupristina altissima Bala. & Abd., 1981	*F. altissima*	pollinator	2014-2015	4	MK473970	MK495091	MK559358
					MK473971		MK543367
					MK473972		MK543368
					MK473973		MK543369
Eupristina cyclostigma Wiebes, 1992	*F. stricta*	pollinator	2008-2018	1	**GQ367607**	**GQ367804**	**GQ367900**
					MK474057		
Eupristina koningsbergeri Grandi, 1916	*F. benjamina*	pollinator	2014-2015	6	MK473974	MK495092	MK543373
					MK473975		MK543374
					MK473976		MK543375
					MK473977		MK543376
							MK543377
							MK543378
Eupristina sp. 1	*F. altissima*	cheater	2014-2015	11	MK473978	-	-
					MK473979		
					MK473980		
					MK473981		
					MK473982		
					MK473983		
					MK473984		
					MK473985		
					MK473986		
					MK473987		

Family, subfamily and species	Host *Ficus* species	Ecological role	Collection period	Number of individuals	18S	28S	*COI*
					MK473988		
Eupristina sp. 2	*F. maclellandii*	pollinator	2008-2018	3	MK473989	MK495093	-
					MK473990	MK495094	
					MK473991	MK495095	
Eupristina sp. 3	*F. microcarpa*	cheater	2014-2015	8	MK473992	MK495096	MK543379
					MK473993	MK495097	MK543380
					MK473994	MK495098	MK543381
					MK473995	MK495099	MK543382
					MK473996	MK495100	MK543383
					MK473997	MK495101	MK543384
					MK473998	MK495102	MK543385
						MK495103	MK543386
Eupristina verticillata Waterston,1921	*F. microcarpa*	pollinator	2014-2015	8	MK474006	MK495110	MK543392
					MK474007	MK495111	MK543393
					MK474008	MK495112	MK543394
					MK474009	MK495113	MK543395
					MK474010	MK495114	MK543396
					MK474011	MK495115	MK543397
					MK474012	MK495116	MK543398
							MK543399
Hederagaon sp.	*F. pubigera*	pollinator	2008-2018	1	MK474054	-	MK559431
Maniella sp. 1	*F. glaberrima*	pollinator	2008-2018	6	MK474041	MK495134	MK543419
					MK474042	MK495135	MK543420
					MK474043	MK495136	MK543421
					MK474044	MK495137	MK543422
					MK474045	MK495138	MK543423
					MK474046		MK543424
Maniella sp. 2	*F. talboti*	pollinator	2008-2018	7	MK473999	MK495104	MK559359
					MK474000	MK495105	MK543387
					MK474001	MK495106	MK543388
					MK474002	MK495107	MK543389
					MK474003	MK495108	MK543390
					MK474004	MK495109	MK543391
					MK474005		
Platyscapa quadraticeps (Mayr, 1885)	*F. religiosa*	pollinator	2008-2018	5	MK474033	MK495131	MK543412
					MK474034	MK495132	MK543413
					MK474035	MK495133	MK559360
							MK543414

Family, subfamily and species	Host *Ficus* species	Ecological role	Collection period	Number of individuals	18S	28S	*COI*
							MK543415
Platyscapa sp. 1	*F. concinna*	pollinator	2014-2015	5	MK474028	MK495127	MK543408
					MK474029	MK495128	MK543409
					MK474030	MK495129	MK543410
					MK474031	MK495130	MK543411
					MK474032		
Umagaon sp.	*F. orthoneura*	pollinator	2008-2018	3	MK474047	MK495139	MK543425
					MK474048	MK495140	MK543426
					MK474049	MK495141	MK543427
Waterstoniella sp.	*F. curtipes*	pollinator	2014-2015	4	MK474050	MK495142	MK543428
					MK474051		MK543429
					MK474052		MK543430
					MK474053		MK543431
**Kradibiinae**							
Ceratosolen emarginatus Mayr, 1906	*F. auriculata*	pollinator	2014-2015	6	MK473930	MK495055	MK543332
					MK473931	MK495056	MK543333
					MK473932	MK495057	MK543334
					MK473933	MK495058	MK543335
					MK473934	MK495059	MK543336
					MK473935	MK495060	MK543337
Ceratosolen emarginatus Mayr, 1906	*F. oligodon*	pollinator	2014-2015	10	MK473936	MK495061	MK543338
					MK473937	MK495062	MK543339
					MK473938	MK495063	MK543340
					MK473939	MK495064	MK543341
					MK473940	MK495065	MK559357
					MK473941	MK495066	MK543342
					MK473942	MK495067	MK543343
					MK473943	MK495068	MK543344
					MK473944	MK495069	MK543345
					MK473945	MK495070	MK543346
Ceratosolen fusciceps (Mayr, 1885)	*F. racemosa*	pollinator	2014-2015	5	MK473946	MK495071	MK543347
					MK473947	MK495072	MK543348
					MK473948	MK495073	MK543349
					MK473949		MK543350
					MK473950		MK543351
Ceratosolen gravelyi Grandi, 1916	*F. semicordata*	pollinator	2014-2015	5	MK473951	MK495074	MK543352
					MK473952	MK495075	MK543353
					MK473953	MK495076	MK543354

Family, subfamily and species	Host *Ficus* species	Ecological role	Collection period	Number of individuals	18S	28S	*COI*
					MK473954	MK495077	MK543355
					MK473955	MK495078	MK543356
Ceratosolen hewitti Waterston, 1921	*F. fistulosa*	pollinator	2008-2018	1	**JN102908**	**JN102690**	**JN103253**
							MK543432
Ceratosolen solmsi martchali Mayr, 1906	*F. hispida*	pollinator	2014-2015	5	MK473961	MK495084	MK543362
					MK473962	MK495085	MK543363
					MK473963	MK495086	MK543364
					MK473964	MK495087	MK543365
					MK473965		
Ceratosolen sp. 1	*F. heterostyla*	pollinator	2014-2015	5	MK473956	MK495079	MK543357
					MK473957	MK495080	MK543358
					MK473958	MK495081	MK543359
					MK473959	MK495082	MK543360
					MK473960	MK495083	MK543361
Ceratosolen sp. 2	*F. prostrata*	pollinator	2008-2018	1	MK473966	**JN102677**	MK543366
Ceratosolen sp. 3	*F.* sp.	pollinator	2014-2015	3	MK473967	MK495088	MK543367
	(*F. auriculata* complex)				MK473968	MK495089	MK543368
					MK473969	MK495090	MK543369
Cerasotolen sp. 4	*F. squamosa*	pollinator	2014-2015	2	**JN102915**	**JN102695**	**JN103260**
					MK474055		
					MK474056		
Kradibia ruthefordi (Waterston, 1920)	*F. tinctoria*	pollinator	2014-2015	7	MK474021	MK495120	**-**
					MK474022	MK495121	
					MK474023	MK495122	
					MK474024	MK495123	
					MK474025	MK495124	
					MK474026	MK495125	
					MK474027	MK495126	
Kradibia subulatae (Hill, 1969)	*F. subulata*	pollinator	2008-2018	0	**JN102920**	**JN102699**	**JN103265**
**Sycophaginae**							
Sycophaga cunia	*F. semicordata*	galler	2014-2015	5	MK495037	MK495217	MK530774
					MK495038	MK495218	MK530775
					MK495039	MK495219	MK530776
					MK495040	MK495220	MK530777
					MK495041	MK495221	MK530778
Sycophaga mayri	*F. racemosa*	galler	2014-2015	5	MK495042	MK495222	MK530779
					MK495043	MK495223	MK543434

Family, subfamily and species	Host *Ficus* species	Ecological role	Collection period	Number of individuals	18S	28S	*COI*
					MK495044	MK495224	
					MK495045	MK495225	
					MK495046		
Sycophaga testacea (Mayr, 1885)	*F. racemosa*	galler	2014-2015	5	MK495047	MK495226	MK530780
					MK495048	MK495227	MK530781
					MK495049	MK495228	MK530782
					MK495050	MK495229	MK530783
					MK495051	MK495230	MK530784
**Pteromalidae**							
**Epichrysomallinae**							
Acophila sp. 1	*F. altissima*	galler	2014-2015	5	MK494956	MK495150	MK530718
					MK494957	MK495151	MK530719
					MK494958	MK495152	MK530720
					MK494959		
					MK494960		
Acophila sp. 2	*F. benjamina*	galler	2014-2015	4	MK494949	MK495143	MK530713
					MK494950	MK495144	MK530714
					MK494951	MK495145	
					MK494952	MK495146	
Acophila sp. 3	*F. concinna*	galler	2014-2015	1	MK494953	MK495147	MK530715
Acophila sp. 4	*F. maclellandii*	galler	2008-2018	2	MK494954	MK495148	MK530716
					MK494955	MK495149	MK530717
Acophila sp. 5	*F. stricta*	galler	2008-2018	1	MK495054	MK495232	MK530787
Camarothorax sp.	*F. concinna*	galler	2014-2015	1	MK494971	MK495161	MK543435
Neosycophila omeomorpha Grandi,1923	*F. tinctoria*	galler	2014-2015	6	MK494972	MK495162	MK530728
					MK494973	MK495163	MK530729
					MK494974	MK495164	MK530730
					MK494975	MK495165	MK530731
					MK494976	MK495166	MK530732
					MK494977	MK495167	MK530733
Odontofroggatia corneri Wiebes,1980	*F. microcarpa*	galler	2014-2015	5	MK494978	MK495168	MK530734
					MK494979	MK495169	MK530735
					MK494980	MK495170	MK530736
					MK494981	MK495171	MK530737
					MK494982	MK495172	
Odontofroggatia galili Wiebes,1980	*F. microcarpa*	galler	2014-2015	1	MK494983	MK495173	MK530738
Sycobia sp. 1	*F. altissima*	galler	2014-2015	1	MK494961	MK495153	MK530721
Sycobia sp. 2	*F. benjamina*	galler	2014-2015	2	MK494963	-	-

Family, subfamily and species	Host *Ficus* species	Ecological role	Collection period	Number of individuals	18S	28S	*COI*
					MK494964		
Sycobia sp. 3	*F. benjamina*	galler	2014-2015	5	MK494965	MK495155	MK530723
					MK494966	MK495156	MK530724
					MK494967	MK495157	MK530725
					MK494968	MK495158	MK530726
					MK494969	MK495159	
Sycobia sp. 4	*F. benjamina*	galler	2014-2015	1	MK494970	MK495160	MK530727
Sycobia sp. 5	*F. curtipes*	galler	2014-2015	1	MK494962	MK495154	MK530722
Sycophilomorpha sp.	*F. altissima*	galler	2014-2015	1	MK494984	-	-
**Otitesellinae**							
Lipothymus sp.	*F. curtipes*	secondary galler	2014-2015	1	MK495053	-	MK530786
Micranisa ralianga Mathew & Balakrishnan, 1981	*F. altissima*	galler	2014-2015	2	MK495011	MK495194	MK530759
					MK495012		
					MK495013		
					MK495014		
Micranisa sp. 1	*F. altissima*	galler	2014-2015	6	MK495005	MK495193	MK530758
					MK495006		
					MK495007		
					MK495008		
					MK495009		
					MK495010		
Micranisa sp. 2	*F. concinna*	galler	2014-2015	1	MK495015	MK495195	MK530760
Micranisa sp. 3	*F. curtipes*	galler	2014-2015	1	MK494985	MK495174	MK530739
Micranisa sp. 4	*F. maclellandii*	galler	2008-2018	6	MK494986	MK495175	MK530740
					MK494987	MK495176	MK530741
					MK494988	MK495177	MK530742
					MK494989	MK495178	MK530743
					MK494990	MK495179	MK530744
					MK494991	MK495180	MK530745
Micranisa sp. 5	*F. microcarpa*	galler	2014-2015	7	MK494992	MK495181	MK530746
					MK494993	MK495182	MK530747
					MK494994	MK495183	MK530748
					MK494995	MK495184	MK530749
					MK494996	MK495185	MK530750
					MK494997	MK495186	MK530751
					MK494998		
Micranisa sp. 6	*F. talboti*	galler	2008-2018	6	MK494999	MK495187	MK530752

Family, subfamily and species	Host *Ficus* species	Ecological role	Collection period	Number of individuals	18S	28S	*COI*
					MK494500	MK495188	MK530753
					MK494501	MK495189	MK530754
					MK494502	MK495190	MK530755
					MK494503	MK495191	MK530756
					MK494504	MK495192	MK530757
Walkerella benjamini Joseph,1957	*F. benjamina*	galler	2014-2015	6	MK495026	MK495206	MK530768
					MK495027	MK495207	MK530769
					MK495028	MK495208	
					MK495029	MK495209	
					MK495030	MK495210	
						MK495211	
Walkerella curtipedis Ma & Yang	*F. curtipes*	galler	2014-2015	1	MK495016	MK495196	MK530761
Walkerella microcapae Bouček,1993	*F. microcarpa*	galler	2014-2015	6	MK495031	MK495212	MK530770
					MK495032	MK495213	MK530771
					MK495033	MK495214	MK530772
					MK495034	MK495215	MK530773
					MK495035	MK495216	
					MK495036		
Walkerella nigrabdomina Ma & Yang	*F. talboti*	galler	2008-2018	5	MK495020	MK495200	MK530764
					MK495021	MK495201	MK530765
					MK495022	MK495202	MK530766
					MK495023	MK495203	MK543433
					MK495024	MK495204	
Walkerella sp. 1	*F. altissima*	galler	2014-2015	1	MK495025	MK495205	MK530767
Walkerella sp. 2	*F. glaberrima*	galler	2008-2018	3	MK495017	MK495197	MK530762
					MK495018	MK495198	MK530763
					MK495019	MK495199	
**Sycoecinae**							
Diaziella yangi van Noort & Rasplus 2006	*F. curtipes*	secondary galler	2014-2015	1	MK495052	MK495231	MK530785
**OUTGROUP**							
Ficomila sp.					**GQ367656**	**GQ367850**	**GQ367946**
Megastigmus sp.					**GQ367582**	**GQ367780**	**GQ367876**
Sycophaga cyclostigma Waterston, 1916					**GQ367651**	**GQ367845**	**GQ367941**
Trichogramma evanescens Westwood, 1833					**GQ367672**	**GQ367865**	**GQ367960**
